# Sweeping analysis of transcript profile in dengue virus serotype 3 infection and antibody-dependent enhancement of infection

**DOI:** 10.1080/21505594.2021.1996072

**Published:** 2021-11-08

**Authors:** Mingwang Long, Yue Pan, Junying Chen, Fan Jia, Han Wang, Daiying Li, Kai Feng, Lingmei Yan, Xiaodan Wang, Xuelei Ning, Lijuan Qiu, Juan Zhang, Qiangming Sun

**Affiliations:** a Institute of Medical Biology, Chinese Academy of Medical Sciences, and Peking Union Medical College, Kunming, Peoples Republic of China; b Yunnan Key Laboratory of Vaccine Research & Development on Severe Infectious Diseases, Kunming, PR China; c Yunnan Key Laboratory of Vector-borne Infectious Disease, Kunming, Peoples Republic of China; d Kunming Medical University, Kunming, Peoples Republic of China; e Yunnan University, Kunming, Peoples Republic of China

**Keywords:** DENV-3 (dengue virus serotype 3), ade (antibody-dependent enhancement), high-throughput sequencing, rna-seq, differentially expressed rnas (de rnas), rnas expression profile, denv-host interactions, immune system, viral infectious diseases, interferon-stimulated genes

## Abstract

Dengue virus infection mainly causes dengue hemorrhagic fever (DHF) and/or dengue shock syndrome (DSS). However, ADE (antibody-dependent enhancement) is one of the main pathogenic factors, and its pathogenic mechanism has not been fully elucidated. Recently, with the development of high-throughput sequencing, an increased number of RNAs have been confirmed to play a vital regulatory role in the process of virus infection. However, there is a lack of research on dengue virus infection and ADE. In this study, we used RNA-Seq to detect differentially expressed RNAs (DE RNAs) profiles in mock-infected, DENV-3-infected, and ADE-infected THP-1 cells. Firstly, we found 69 circRNAs, 259 miRNAs, and 18 mRNAs were differentially expressed in THP-1 vs DENV-3. In THP-1 vs ADE, 94 circRNAs, 263 miRNAs, and 111 mRNAs were differentially expressed. In DENV-3 vs ADE, 68 circRNAs, 105 miRNAs, and 94 mRNAs were differentially expressed. Functional enrichment analysis of these DE RNAs mainly focused on immune system, viral infectious diseases, cytokine-cytokine receptor interactions, and NOD/RIG-I-like receptor signaling pathways. In DENV-3 vs ADE, notably, the expression of HBB was up-regulated, which was a Fcγ Receptor-mediated phagocytosis protein. Additionally, we predicted the encoding ability of DE circRNAs, and it was found that a small peptide was encoded by novel_circ_001562 and that its amino acid sequence was consistent with that of DDX60L, which is a class of interferon-stimulated genes. Finally, we constructed the ceRNA regulatory network pathway. Therefore, our study provides a new strategy for further investigation on DENV-host interactions.

## Introduction

Dengue virus belongs to the *Flavivirus genus* of the *Flaviviridae family*. It is a type of arbovirus, which has been spread by *Aedes albopictus* and *Aedes aegypti*, and is widespread in tropical and subtropical regions [1]. Dengue virus is a type of enveloped, single positive-stranded RNA (+ssRNA) virus with a whole-genome length of approximately 10.7kb [1]. The viral genome encodes a polyprotein with a whole length of 3,500 amino acids, which is cleaved by the host protease to form 10 proteins, including 7 nonstructural proteins and 3 structural proteins [2]. The structural proteins mainly include Capsid protein (C protein), Envelope protein (E protein), and Pre-Membrane protein (PrM protein). The PrM protein can be cleaved by the host furin enzyme to form a mature M protein. The nonstructural proteins include nonstructural proteins 1 (NS1), nonstructural proteins 2A (NS2A), nonstructural proteins 2B (NS2B), nonstructural proteins 3 (NS3), nonstructural proteins 4 (NS4), nonstructural proteins 5A (NS5A), and nonstructural proteins 5B (NS5B). According to the antigenic difference of E protein, dengue virus can be divided into 4 sub-types: DENV-1, DENV-2, DENV-3, and DENV-4. Most of the infected people are asymptomatic or have only mild symptoms. About 25% of the infected people have fever (known as dengue fever) and abnormal blood biochemical indicators [3]. Only a few infected people have severe symptoms, e.g., dengue hemorrhagic fever (DHF), dengue shock syndrome (DSS), vascular leakage syndrome, coagulation dysfunction, and multiple organ damage [3]. Most severely infected people are more common in secondary infections or multiple infections. Because the first infection can stimulate the immune system to produce high levels of neutralizing antibodise, it can effectively protect against homotype and heterotype dengue virus infections over a period of time. However, as the neutralizing antibody titer drops to the sub-neutralizing antibody titer, it may promote homotype or heterotype dengue virus infection, thereby inducing immune system disorders, leading to serious illnesses [4,5]. This phenomenon is called ADE (antibody-dependent enhancement), and it was first proposed by Halstead and colleagues: when cross-antibody or sub-neutralizing antibody, the virus and antibody form a complex and promote the virus infection process through FCγR II. This phenomenon is found in dendritic cells, macrophages, and monocytes. Therefore, ADE is a receptor-mediated process that promotes viral infection and can escape the host’s antiviral immune response, leading to an imbalance of the inflammatory response, inflammatory cytokine storms, vascular leakage, and immune response imbalance that aggravate the process of disease [6,7]. With the development and application of RNA-Seq, it is found that about 80% of the genome of the host gene does not participate in the transcription and translation process, but it plays a vital regulatory mechanism in the interaction between virus and host [8]. Recently, an increased number of non-coding RNAs have been reported on the regulation mechanism of various viral infections and interactions between host and virus [9–12]. In flavivirus infections, some non-coding RNAs functions have also been reported. For example, microRNA-30e* inhibits the infection process of DENV-2 via regulating the NF-κB/IFN signaling pathway [13]. lncRNA (long non-coding RNA) Malat1 is up-regulated during flavivirus infection, and regulates the host’s response to viral infection through the PERK signaling pathway [14]. Single-cell sequencing of body fluid samples from patients infected with dengue virus revealed that some RNAs can predict the development of illness [15]. In addition, microRNA-155 can regulate the process of antiviral and inflammatory response in West Nile virus infection [16]. As a type of RNAs with special structure, circRNA mainly regulate the expression level of genes by adsorbing microRNAs to form a ceRNA regulatory network. This phenomenon is found in many diseases, such as tumor [17,18], cardiovascular disease [19], infectious disease [20], and obesity [21]. However, at the level of RNAs, the pathogenesis of dengue virus infection and ADE, and the mechanism of the interaction between the virus and the host remain unclear. Therefore, we analyzed the DE RNA profiles and function in mock-infected, DENV-3-infected, and ADE-infected THP-1 cells and then analyzed whether circRNAs can encode small peptide amino acid sequences and their structures. The regulatory network of ceRNAs was built to supply a new strategy for investigation on the molecular mechanism of dengue virus infection and ADE.

## Materials and methods

### Cell and DENV-3

DENV-3 strain (GenBank number: MW426463) was from our department. RPMI-1640 medium containing streptomycin (100 μg/ml), penicillin (100 U/ml) and fetal bovine serum (10%) was used for THP-1 and C6/36 cell culture. THP-1 was used to establish DENV-3 infection and ADE models. C6/36 cells were used for virus amplification.

### DENV-3 infection and ADE models in vitro

The Anti- Dengue- II PrM antibody (AB41473) and DENV-3 were used to establish DENV-3 infected THP-1 and ADE models at multiplicity of infection of 0.3, in accordance with our previous study [22].

### Western blotting (WB)

Mock-infected, ADE-infected, and DENV3-infected THP-1 cells were lysed by RIPA to extract total protein. The total protein was separated by SDS-PAGE, then transferred to PVDF membrane by electroporation, and blocked with skim milk (5%) at room temperature for 90 minutes. Then it was incubated with the primary antibody (Anti-DENV-III E protein antibody) overnight at four centigrade degree, and washed three times with PBST, 5 min each time. It was then incubated with the secondary antibody for 2 h under room temperature. Finally, the DENV-3 E protein signals can be visualized.

### Immunofluorescence (IF)

The Anti-DENV-III E protein Antibody as primary antibody and Cy3-conjugated secondary antibody (AB97035) were used to stain the virus E protein in the cytoplasm, and DAPI (US EVERBRIGHT INC) was used to stain the nucleus. Finally, the DENV-3 E protein signal and the nucleus were observed under a confocal microscope.

### RNA extraction and RNA analysis

#### RNA extraction

Intracellular total RNA was extracted using Trizol (Invitrogen) 48 h after mock-infection, DENV3-infection, and ADE-infection, in accordance with the manufacturer’s instruction.

#### Detection of viral nucleic acid copy number by qRT-PCR

According to our previous experiment [22], DENV-3-qRT-PCR Primers and Plasmid-DENV-3-qRT-PCR Primers **(Table S1.)** were designed to target the DENV-3 PrM and DENV-3 PrM within the standard, respectively. Then, the number of viral nucleic acid copies was converted according to the standard curve. The qRT-PCR reagents are TB Green Premix Ex Taq^TM^ II (TAKARA RR820A), in accordance with the manufacturer’s instruction.

#### Identification of circRNAs and DE circRNAs

The intracellular total RAN was digested to degrade linear RNA with RNaseR, and then purified using RNeasy MinElute Cleanup Kit (Qiagen) . These digestive products were sequenced by PCR amplification, and then RNAs sequence was compared with Find_Circ. Differential analysis was performed for all CircRNAs using edgeR package with a fold change ≥2. *p* value <0.05 indicated DE CircRNAs.

#### ORFs Prediction and IRES prediction

The two software, which are cORF pipeline [23] script and IRES finder [24], were used to predict ORF and IRES to determine if these DE CircRNAs can encode the polypeptide.

#### Identification of miRNA and differentially expressed miRNAs

All of the clean data were compared with miRBase database (Release 22). All miRNAs were analyzed using edgeR package with a fold change ≥2, and *p* value <0.05 indicated DE miRNAs.

#### Functional enrichment analysis

Gene Ontology Functional Enrichment Analysis (GO, http://www.geneontology.org/) and Kyoto Encyclopedia of Genes and Genomes Functional Enrichment Analysis (KEGG, https://www.kegg.jp/)

#### Construction of differentially expressed “circRNAs-miRNAs-mRNAs” regulatory network

In order to foretell DE miRNAs sponge with DE circRNAs and mRNAs, the miRTarBase (version 6.1) was used to find differentially expressed miRNAs interacting with DE mRNAs and circRNAs. The correlation of DE circRNAs-miRNAs and DE miRNAs-mRNA can be visualized via Cytoscape (https://cytoscape.org/).

#### Differentially expressed circRNAs, microRNAs and mRNAs were identified by qRT-PCR analysis

In order to verify the accuracy of RNA-Seq. Fisrtly, we using PrimeScript^TM^ reagent kit with gDNA Eraser (TAKARA RR047A) and PCR instrument (Bio-Rad) to reverse transcription reaction, according to the instructions. Then, we using TB Green Premix Ex Taq^TM^ II (TAKARA RR820A) and Fluorescence quantitative PCR instrument (Bio-Rad) to detect the RNA expression level, according to the instructions.The RT-qPCR was used to investigate the relative levels of DE RNAs. The primers for those DE RNAs and GAPDH are shown in **Table S1**. MicroRNAs needs to replace the random primers in Takara RR047A with specific primers (**Table S1)** for specific stem ring detection to reverse transcription reaction.

#### Statistical analysis

SPSS17.0 software was used to analyze the mean value difference after three independent repeated trials in different groups. Through one-way ANOVA of multiple groups, it was determined that the difference was statistically significant when *p* < 0.05. Single, double and three asterisks, and * indicate statistical significance (* *p* < 0.05; * *p* < 0.01; * *p* < 0.001).

## Results

### Establishment of a model of ADE in DENV-3 infected THP-1 cells

**Figure 1.**
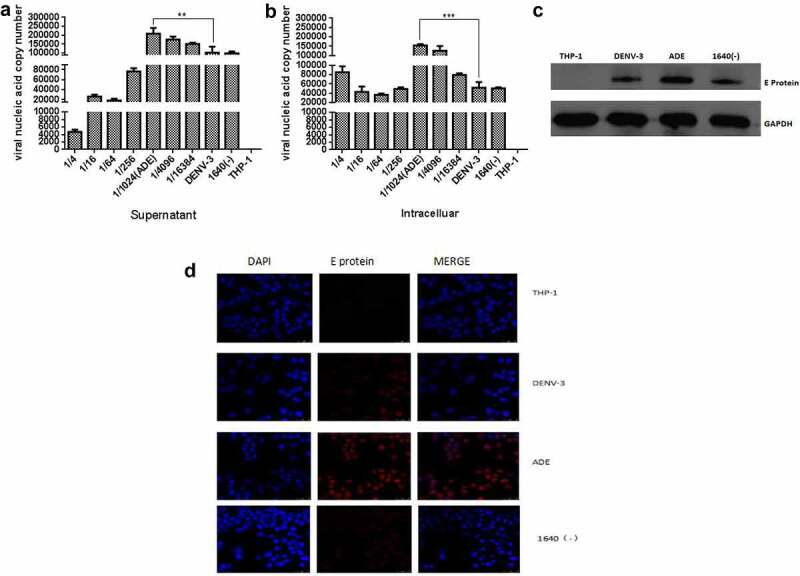
Establishing a model of ADE in DENV-3 infected THP-1 cells

Currently, it is considered that ADE is mediated by FCγR [25], and ADE models of dengue virus infection have been successfully established in THP-1, U937, and K562 cells [26]. Based on our previous research [22], Anti-DENV-II PrM antibody and DENV-3 were used to set up DENV-3 infection and ADE models in THP-1 cells. At 48 h post-infection, the qRT-PCR was used to detect the DENV-3 genome RNA in the supernatant and cells, and the Western blotting and Immunofluorescence were used to detect the intracellular DENV-3 E protein. As shown in Figures 1(a,b), different dilutions of anti-DENV-II PrM antibodies promoted or inhibited DENV-3 infection compared with DENV-3 direct infection in THP-1 cells. When the anti-DENV-II PrM was diluted by 1/1,024, the copy number of viral nucleic acid in the cell and supernatant was significantly higher compared to DENV-3 direct infection and other dilutions. The same results can also be observed in Figures 1(c,d). At a dilution of 1/1,024, the DENV-3 E protein was found to be significantly higher than that of direct infection with DENV-3. Therefore, DENV-3 can infect THP-1, and the anti-DENV-II PrM antibody at a dilution of 1/1,024 can establish the ADE model in THP-1.

At 48 h post-infection, supernatant **(A)** and cells **(B)** were collected for total RNA, and then DENV-3 genome RNA was detected using RT-qPCR. Then, THP-1 cells were collected for total protein at 48 h post-infection, the Western blotting (**C)** and Immunofluorescence **(D)** were used to detect intracellular DENV-3 E protein. The 1640(-) represents dilution of DENV-3 with RPMI-1640 medium without Anti- Dengue -II PrM antibody as negative control.

### Transcriptome sequencing data quality assessment

**Figure 2.**
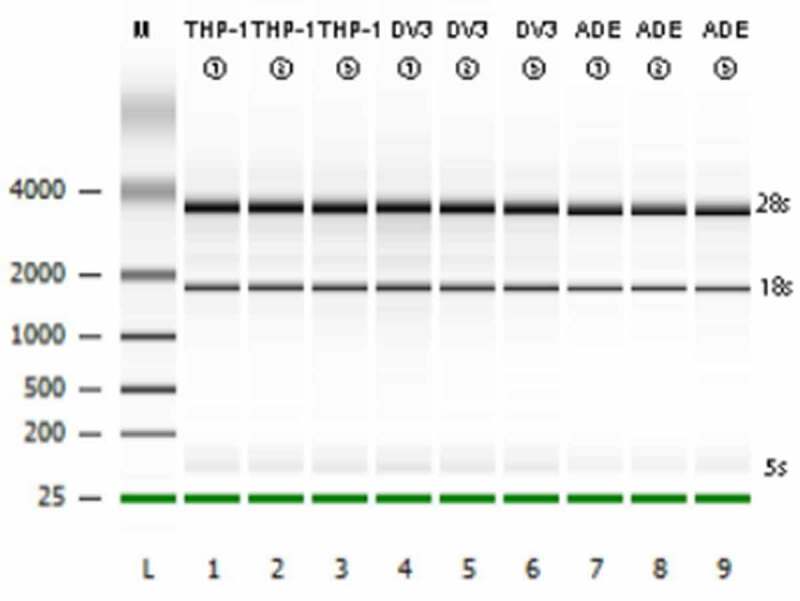
Transcriptome sequencing data quality assessment

Before proceeding with RNA-Seq, agarose gel electrophoresis was used to detect the integrity of the total RNA extracted from the cells. According to Bioanalyzer plot, as shown in Figure 2(a), S28, S18, and S5 bands were clearly visible, indicating that the integrity of the RNA was good and fulfilled the requirements of sequencing. The transcriptome sequencing on the IIIumina 6000 harvested at least 6.0Gb of clean data per sample, which can be used for further analysis after quality control. The clean data of all samples were above 99.77% (Table 1), and the GC content of the cleaning data of all samples were 46.6–47.58% (Table 1). These high-quality clean data were compared with the reference human genome (RHG, Ensembl_release102. GenBank number: GCA-000001405.28). Approximately 96.22% to 97.05% of the clean reads (CRs) were successfully matched to the RHG, and 92.69% to 93.8% of the CRs were uniquely matched to the RHG (Table 1). Thus, it can be seen that the completeness and reliability of the sequencing results are high, and these results can be used for further research.

**Table 1.** Transcriptome sequencing data quality assessment

Sample	Raw Datas (reads)	CleanData (%)	Q30 (%)	GC (%)	Total Mapped (%)	Multiple Mapped (%)	Uniquely Mapped (%)
**THP-1-1**	80,163,930	99.79	93.98	47.12	96.47	3.47	93.00
**THP-1-2**	80,042,926	99.77	94.05	47.26	96.22	3.53	92.69
**THP-1-3**	79,661,264	99.80	94.06	46.71	96.50	3.35	93.15
**DENV-3-1**	77,621,938	99.80	94.35	47.33	96.76	3.55	93.21
**DENV-3-2**	81,393,494	99.79	94.03	47.22	96.60	3.48	93.12
**DENV-3-3**	83,677,460	99.81	94.16	47.58	96.68	3.53	93.15
**ADE-1**	95,949,024	99.80	93.77	47.10	96.73	3.43	93.30
**ADE-2**	82,128,672	99.80	93.34	47.14	96.54	3.31	93.23
**ADE-3**	89,317,446	99.81	94.11	46.60	97.05	3.25	93.80

The integrity of RNA was detected by gel agarose gel electrophoresis **(A)**.

### Analysis of DE circRNAs in mock-infected, DENV3-infected, and ADE-infected THP-1 cells

**Figure 3.**
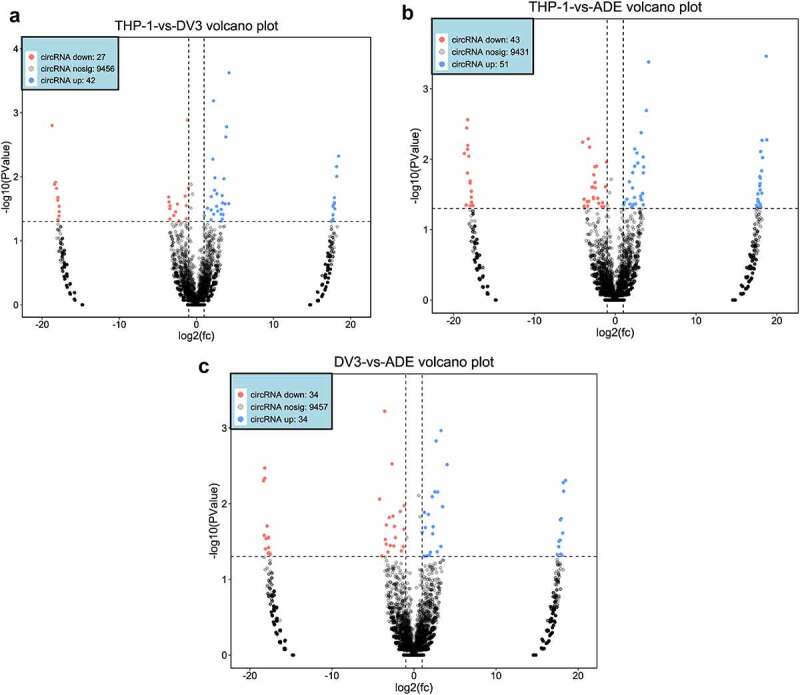
DE circRNAs expression profile

Using rRNA deletion samples from the RNA of mock-infected, DENV3-infected, and ADE-infected THP-1 cells, circRNA sequencing was performed. An average of about 79 million, 80 million, and 89 million CRs were found in the THP-1, DENV-3, and ADE groups, respectively (Table 1). 78.9% of DE circRNAs were exons, 4.04% were introns, and 1.09% were located at the intergenic region (Figure S1A, Supplementary material 1). Then, it was found that the size of most DE circRNAs was 100–3,000 nt. The predicted splicing lengths of DE circular RNAs were 100–1,000nt (Figure S1B, Supplementary material 1). These differentially expressed circRNAs were located at chromosomes, such as chromosome 17, chromosome 5, chromosome 3, chromosome 2, and chromosome 1 (Figure S1C, Supplementary material 1). A total of 69, 94, and 68 DE circRNAs were identified in THP-1 vs DENV-3, THP-1 vs ADE, and DENV-3 vs ADE, respectively. Among those, in THP-1 vs DENV-3 (Figure 3(a)), 42 DE circRNAs expressions were increased and 27 DE circRNAs expressions were reduced. In THP-1 vs ADE (Figure 3(b)), 51 DE circRNAs expressions were increased and 43 DE circRNAs expressions were reduced. In DENV-3 vs ADE (Figure 3(c)), 34 DE circRNAs expressions were increased and 34 DE circRNAs expressions were decreased. In addition, we also performed a heatmap analysis on all DE circRNAs (Figure S1D, Supplementary material 1). In DENV-3 vs ADE, one half of those dysregulated DE circRNAs were down-regulated and the other half were up-regulated.

DE circRNAs chart of THP-1 VS DENV-3 **(A)**, THP-1 VS ADE **(B)** and DENV-3 VS ADE **(C)**. Up-regulated circRNAs were blue and down-regulated circRNAs were red.

### Analysis of DE mRNAs and DE miRNAs in mock-infected, DENV-3-infected, and ADE-infected THP-1 cells

**Figure 4.**
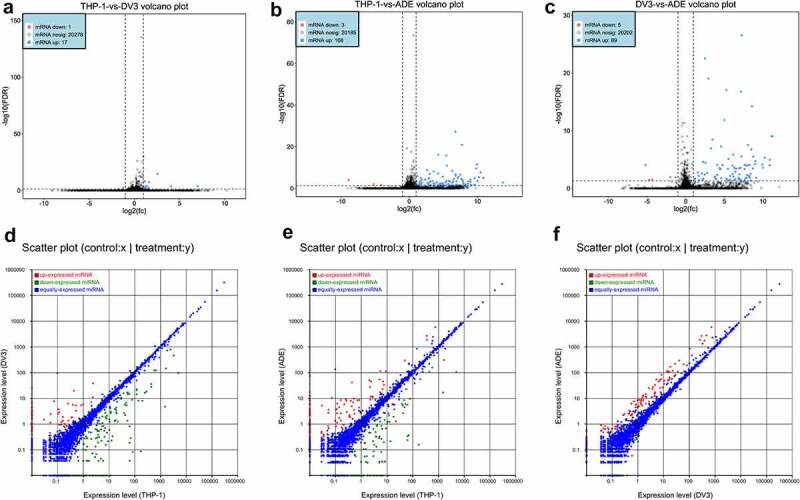
Differentially expressed mRNAs and miRNAs profiles

We detected 18, 111 and 94 DE mRNAs in THP-1 vs DENV-3, THP-1 vs ADE, and DENV-3 vs ADE, respectively. Among those, 17 DE mRNAs were up-regulated in THP-1 vs DENV-3, and 1 was down-regulated (Figure 4(a)). A total of 108 DE mRNAs were up-regulated in THP-1 vs ADE, and 3 were down-regulated (Figure 4(b)). In DENV-3 vs ADE, 89 DE mRNAs expressions were increased and 5 were reduced (Figure 4(c)). In addition, a heatmap analysis was performed on all DE mRNAs, and the results are shown in Figure S2A (Supplementary material 1) . Therefore, in DENV-3 infection and ADE, most of the DE mRNAs were up-regulated. Additionally, the sequencing data showed 252, 254, and 96 DE miRNAs were abnormally expressed in THP-1 vs DENV-3, THP-1 vs ADE, and DENV-3 vs ADE, respectively. Among THP-1 vs DENV-3, 68 DE miRNAs expressions were increased and 184 DE miRNAs expressions were reduced (Figure 4(d)). In THP-1 vs ADE, 123 DE miRNAs expressions were raised, and 131 DE miRNAs expressions were lowered (Figure 4(e)). In DENV-3 vs ADE, 92 DE miRNAs expressions were enhanced and 4 DE miRNAs expressions were decreased (Figure 4(f)). In addition, a heatmap analysis was also performed on all DE miRNAs (Figure S2B, Supplementary material 1). Compared with the DENV-3 vs ADE, most of dysregulated DE miRNAs were down-regulated in the THP-1 vs DENV-3. In the THP-1 vs ADE group, the number of DE miRNAs up-regulated and down-regulated was basically the same.

DE mRNAs in THP-1 VS DENV-3 **(A)**, THP-1 VS ADE **(B)**, and DENV-3 VS ADE **(C)**. Up-regulated mRNAs were blue and down-regulated mRNAs were red. DE miRNAs in THP-1 vs DENV-3 **(D)**, THP-1 vs ADE **(E)** and DENV-3 vs ADE **(F)**. Up-regulated miRNAs were red and down-regulated miRNAs were green.

### Functional enrichment analysis of the DE RNAs

**Figure 5.**
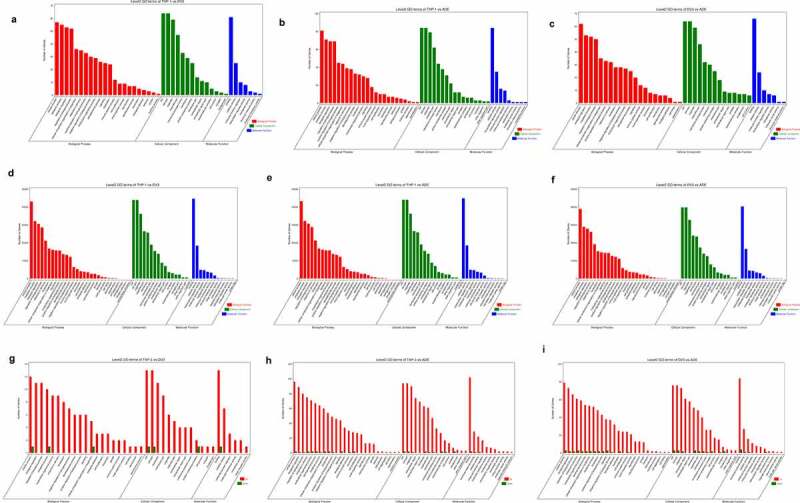
GO and KEGG analysis of DE RNAs

In order to explore the biological function of DE RNAs in mock-infected, DENV-3-infected, and ADE-infected THP-1 cells, we tested the parental genes of differentially expressed RNAs through functional analysis. According to the results of GO analysis, DE circRNAs were mainly enriched in various biological processes (Figures 5(a-c)), such as “metabolic process”, “biological regulation” and “response to stimulation”. Additionally, the enrichment analysis of KEGG pathway showed that many DE circRNAs were mainly enriched in the “immune system” and “immune diseases”, “infectious diseases: viruses” and “cell growth and death” (Figure S3A-S3B, Supplementary material 1). Genes related to the “immune system” were up-regulated in DENV-3 vs ADE. These data indicated that differentially expressed circRNAs were mainly related to “infectious diseases: viral” and “host immune response”. According to the results of GO analysis, differentially expressed miRNAs were mainly enriched in a variety of biological processes, including “cellular processes”, “metabolic processes”, “biological regulation”, “response to stimuli” and “immune system” (Figures 5(d-f)). In addition, KEGG pathway enrichment analysis showed that DE miRNAs were mainly enriched in “apoptosis”, “cytokine-cytokine-receptor interaction” and “NOD/RIG-I-like receptor signaling pathway” (Figure S3D-S3F, Supplementary material 1). These DE miRNAs were mainly involved in “apoptosis” and “NOD/RIG-I-like receptor signaling pathways” in DENV-3 vs ADE. The parental genes of differentially expressed mRNAs were mainly enriched in different biological processes, such as “biological regulation”, “cellular processes”, and “immune system processes” (Figures 5(g-i)). Moreover, many differentially expressed mRNAs were mainly enriched in “Fcγ receptor-mediated phagocytosis (such as HBB)”, “complement and coagulation cascade”, “ECM receptor interaction” and “cell growth and death” according to the results of KEGG pathway enrichment analysis (Figure S5G-S5I, Supplementary material 1). These data confirm that the interaction between dengue virus and host is a complex mechanism, and those RNAs participate in the antiviral mechanism through various ways.

GO analysis of DE circRNAs in THP-1 vs DENV-3 **(A)**, THP-1 vs ADE **(B)**, and DENV-3 vs ADE **(C)**, respectively. GO analysis of DE miRNAs in THP-1 vs DENV-3 **(D)**, THP-1 vs ADE **(E)**, and DENV-3 vs ADE **(F)**. GO analysis of DE mRNAs in THP-1 vs DENV-3 **(G)**, THP-1 vs ADE **(H)**, and DENV-3 vs ADE **(I)**.

### Quantitative real-time PCR analysis and prediction of the small peptide-encoding ability of the DE circRNAs of host genes

**Figure 6.**
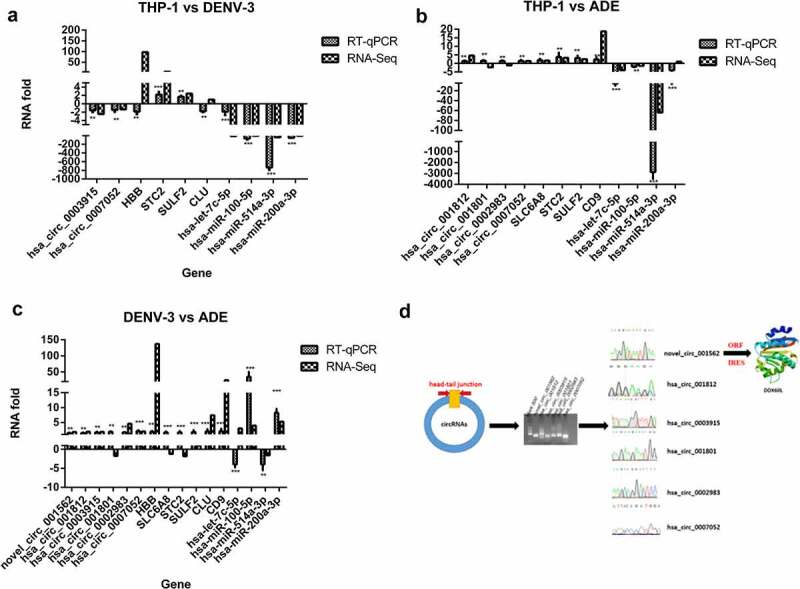
Quantitative real-time PCR analysis and Prediction of small peptide coding ability of circRNA encoded by host gene

In order to confirm the results of RNA-Seq, we used qRT-PCR to verify the results of selected 10, 12, and 16 DE RNAs in THP-1 vs DENV-3, THP-1 vs ADE, and DENV-3 vs ADE, respectively. As shown in (Figures 6(a-c)), RNA-Seq results were verified by RT-qPCR. The hand-tail joint sequence of DE circRNAs was confirmed by PCR and Sanger sequencing (Figure 6(d)). Then, we predicted the ORF (open reading frame) and the IRES (internal ribosome entry site) to confirm whether these 6 differentially expressed circRNAs (novel_circ_001562, hsa_circ_001812, hsa_circ_0003915, hsa_circ_001801, hsa_circ_0002983, and hsa_circ_0007052) can encode small peptides. Except hsa_circ_0002983 (Supplementary material 2), other DE circRNAs can encode small peptides. Among these, the new_circ_001562 was predicted to encode a small peptide. BLAST was performed on the amino acid sequence of the small peptide in NCBI, and then its spatial structure was predicted (Figure 6(d)). It was found that its amino acid sequence was consistent with DDX60L, which belonged to interferon-stimulating genes [27]. In summary, these circRNAs may be involved in the interaction between DENV-3 and the host by regulating the host immune system and encoding polypeptide.

The qRT-PCR confirm DE RNAs in THP-1 vs DENV-3 **(A)**, THP-1 vs ADE **(B)**, and DENV-3 vs ADE **(C)**. Confirmation of DE circRNAs head-jail junction sequence and prediction of small peptide coding ability of novel_circ_001562 **(D)**.

### The regulation network of differentially expressed miRNAs, circRNAs, and mRNAs

**Figure 7.**
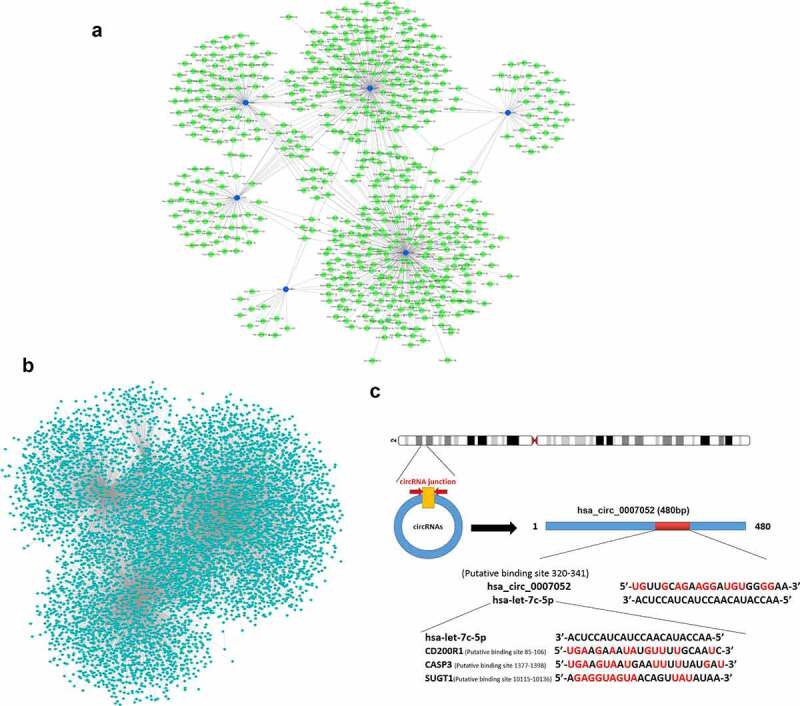
Differentially expressed CircRNAs- miRNAs- mRNAs interaction network

It has been revealed that miRNAs can be competitively sponged by circRNAs, and gene expression is indirectly regulated. To examine the involvement of 6 DE circRNAs (novel_circ_001562, hsa_circ_001812, hsa_circ_0003915, hsa_circ_001801, hsa_circ_0002983, and hsa_circ_0007052), an integrative analysis was performed on the interaction of circRNAs and the target miRNAs. The analysis of miRNAs binding sites in 6 DE circRNAs revealed 661 miRNAs binding sites (Figure 7(a), Supplementary material 3), including the site in hsa_circ_0007052 that can bind with hsa-let-7 c-5p. Next, 19,381 target mRNAs of the 4 differentially expressed miRNAs were predicted, and a miRNAs-mRNAs interaction network was constructed (Figure 7(b), Supplementary material 4), and it was found that hsa-let-7 c-5p can bind with CD200R1, CASP3, SUGT1, etc. CircRNAs (hsa_circ_0007052) may act as ceRNAs, and miRNAs (hsa-let-7 c-5p) is sequestered to interrupt their binding and targeting of mRNAs (CD200R1, CASP3, SUGT1, etc.) (Figure 7(c)). Therefore, circRNA-miRNA-mRNA interactions could play significant roles in the DENV-3-host interaction.

DE CircRNAs- miRNAs regulatory network **(A)**. DE CircRNAs were blue dots, DE miRNAs were green dots. DE miRNAs- mRNAs regulatory network **(B)**. DE miRNAs were green dots, and DE mRNAs were blue dots. CeRNA co-expression network **(C)**. The red base represents complementary pairing with microRNA (hsa-let-7 c-5p).

## Discussion

The function of non-coding RNA has been mainly elucidated in cancer and disease progression [17,18,21]. However, the expression profile, function and mechanism of these non-coding RNAs in viral infections are still unclear. Especially in the process of DENV-3 infection and ADE infection, the characteristics and potential role of non-coding RNA have not been explored. In the current study, the profiles of differentially expressed mRNAs, miRNAs and circRNAs in mock-infected, DENV-3-infected, and ADE-infected THP-1 cells were analyzed. It was found that DENV-3 infection significantly affected the RNA expression profile. In THP-1 vs DENV-3, THP-1 vs ADE, and DENV-3 vs ADE, a total of 69, 94 and 68 DE circRNAs were identified respectively. Among these, in THP-1 vs DENV-3, 42 DE circRNAs expressions were increased and 27 were decreased. In THP-1 vs ADE, 51 DE circRNAs expressions were increased and 43 were decreased. In DENV-3 vs ADE, 34 DE circRNAs expressions were increased and 34 were reduced. Then, we detected 18, 111 and 94 DE mRNAs in THP-1 vs DENV-3, THP-1 vs ADE, and DENV-3 vs ADE, respectively. Among these, most DE mRNAs (94.4%) were up-regulated, and only one was down-regulated in THP-1 vs DENV-3. Similarly, most of DE mRNAs were up-regulated in THP-1 vs ADE (97.3%) and DENV-3 vs ADE (94.7%). There were 252, 254 and 96 DE miRNAs expression abnormalities in THP-1 vs DENV-3, THP-1 vs ADE, and DENV-3 vs ADE respectively. Among THP-1 vs DENV-3, 68 DE miRNAs expressions were increased and 184 DE miRNAs expressions were decreased. In THP-1 vs ADE, the expression of 123 DE miRNAs was up-regulated, and the expression of 131 DE miRNAs was down-regulated. In DENV-3 vs ADE, 92 DE miRNAs were up-regulated and the expressions of 4 DE miRNAs were decreased. It was implied that the regulating mechanism of DE RNAs in the interaction between DENV-3 and host cells may be complex. The activation of innate immunity plays a vital role in the antiviral mechanism, and it is closely related to the regulation of a variety of molecules including RNA. It has been recently reported that some RNAs inhibit host antiviral immunity [11,28,29]. Consistently, the DE circRNAs via GO and KEGG pathways show that these genes are mainly involved in “immune system processes” and “infectious diseases: viruses”. DE mRNAs were found to be involved in “Fcγ receptor-mediated phagocytosis” (such as HBB) and “complement and coagulation cascade”, DE miRNAs were found to be mainly involved in “cytokine-cytokine receptor interaction” and “NOD/RIG-I-like receptor signaling pathway”. In the DENV-3 vs ADE, the expression of HBB was upregulated. Through functional enrichment analysis, it was confirmed that HBB is an Fcγ receptor-mediated phagocytosis protein. This result is consistent with the Fcγ receptor hypothesis of ADE. These data indicate that DE RNAs may regulate DENV-3 infection and ADE infection via antiviral responses, host immune signaling pathways and Fcγ receptor-mediated phagocytosis to promote infection of the dengue virus. Many studies have shown that circRNAs can act as miRNAs sponges to indirectly regulate gene expression. Therefore, we constructed a ceRNA network to reveal the specific role and mechanism of DE circRNAs in DENV-3 and ADE infection. The integration analysis of ceRNA revealed the regulatory function of circRNAs and its specific interaction with mRNAs through the regulation axis of circRNA-miRNA-mRNA in the natural immune response of host cells to DENV-3. We found that circRNA-miRNA-mRNA axis may be involved in the regulation of DENV-3 and ADE infection, such as the hsa_circ_0007052 – hsa-let-7 c-5p – CD200R1/CASP3/ SUGT1 pathway. The CD200R1 signaling pathway can regulate host-pathogen interactions [30], e.g., promoting HSV-1 infection and inflammation through Toll-like receptor 2 (TLR2) [31]. CASP3 inhibits type I interferon through the cleavage of IRF3 and MAVS [32]. SUGT1 regulates HIV-1 infection by stabilizing the positive end of microtubules [33]. Thus, it is speculated that the disorder of differentially expressed circRNAs may be associated with the replication of DENV-3 via antagonizing the inhibition of mRNAs by miRNAs. In addition, many studies have confirmed that circRNAs encode small peptides [34–38]. Considering this evidence, we performed ORF and IRS predictions for 6 differentially expressed circRNAs (novel_circ_001562, hsa_circ_001812, hsa_circ_0003915, hsa_circ_001801, hsa_circ_0002983, hsa_circ_0007052), and found 5 differentially expressed circRNAs (novel_circ_001562, hsa_circ_001812, hsa_circ_0003915, hsa_circ_001801, and hsa_circ_0007052) to determine their encoding potential. Then, we performed BLAST alignment and spatial structure prediction on the amino acid sequence of the small peptide encoded by the novel_circ_001562, and found that it was highly similar to DDX60L, which is an interferon-stimulated gene that can inhibit the replication of hepatitis C virus [27]. Thus, our report firstly reveals DE RNAs profiling and a network of ceRNAs in DENV-3 infection and ADE infection that are involved in the regulation of DENV-3-host interactions, and innovatively investigates mechanisms underlying DENV-3 infection and ADE infection.

## Supplementary Material

Supplemental Material

## Data Availability

In this study, those data are available from the corresponding author based on reasonable requests.
