# Supplementary material for: Sweeping analysis of transcript profile in dengue virus serotype 3 infection and antibody-dependent enhancement of infection
Source: Virulence. 2021 Nov 8;12(1):2764–76. doi: 10.1080/21505594.2021.1996072 (PMC8583062; doi:10.1080/21505594.2021.1996072)
Supplement: Supplemental Material [file KVIR_A_1996072_SM1917.zip › Supplementary material 5.2.pdf]

1. Flow chart of Experiments

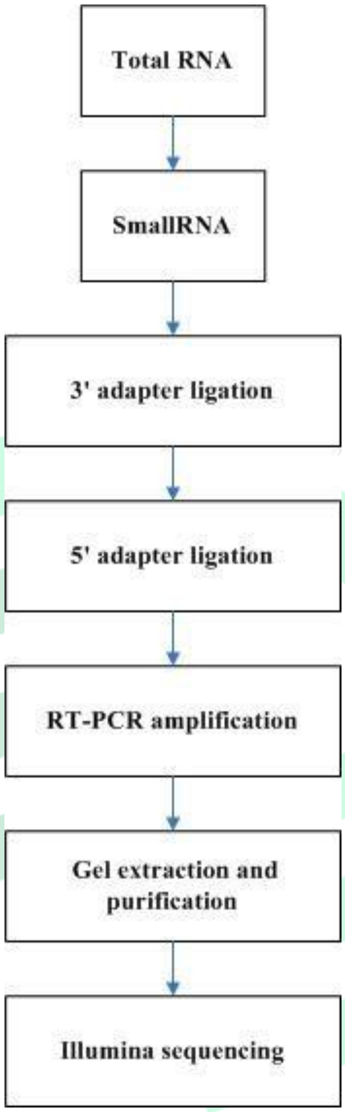

Fig 1. Flow chart of smallRNA experiments

Library construction and sequencing

After total RNA was extracted by Trizol reagent kit (Invitrogen, Carlsbad, CA,USA), the RNA molecules in a size range of 18–30nt were enriched by polyacrylamide gel electrophoresis(PAGE). Then the 3’ adapters were added and the 36-48nt RNAs were enriched. The 5’ adapters were then ligated to the RNAs as well. The ligation products were reverse transcribed by PCR amplification and the 140-160bp size PCR products were enriched to generate a cDNA library and sequenced using Illumina HiSeq Xten by Gene Denovo Biotechnology Co. (Guangzhou, China).

2. Flow chart of bioinformatics analysis

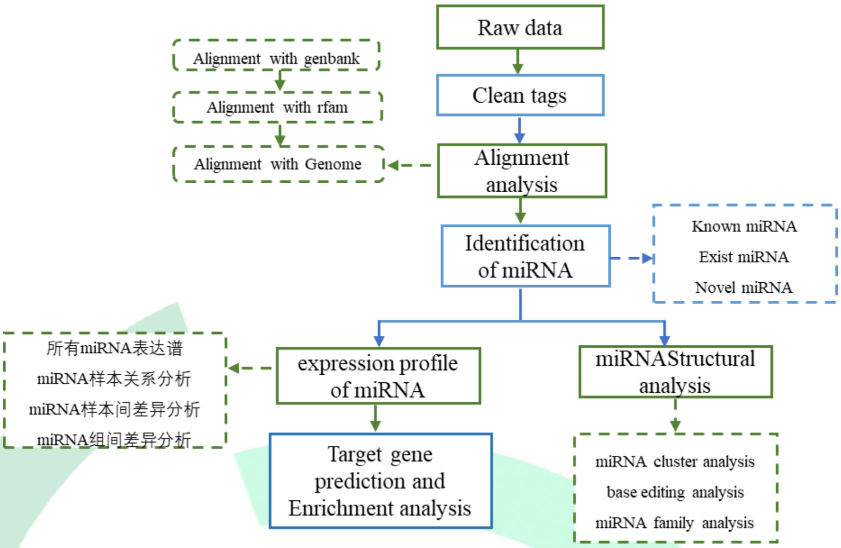

Fig 2. Flow chart of bioinformatics analysis

3 Filtering of Clean Tags

Reads obtained from the sequencing machines included dirty reads containing adapters or low quality bases which would affect the following assembly and analysis. Thus, to get clean tags, raw reads were further filtered according to the following rules:

1) Removing low quality reads containing more than one low quality (Q-value≤20) base or containing unknown nucleotides(N);

2) Removing reads without 3’adapters;

3) Removing reads containing 5’adapters;

4) Removing reads containing 3’ and 5’ adapters but no small RNA fragment between them;

5) Removing reads containing ployA in small RNA fragment;

6) Removing reads shorter than 18nt (not include adapters).

4 Alignment and Identification of small RNA

4.1 Alignment with small RNA in GeneBank

All of the clean tags were aligned with small RNAs in GeneBank database(Release 209.0) to identify and remove rRNA,scRNA,snoRNA,snRNA and tRNA.

4.2 Alignment with small RNA in Rfam

Meanwhile all of the clean tags were aligned with small RNAs in Rfam database(Release 11.0) to identify and remove rRNA,scRNA,sonRNA,snRNA and tRNA.

4.3 Alignment with Genome (exon, intron, repeat sequences)

All of the clean tags were also aligned with reference genome. Those mapped to exons or introns might be fragments from mRNA degradation, so these tags were removed. The tags mapped to repeat sequences were also removed.

4.4 Identification of microRNA (miRNA)

4.4.1 Identification of exist miRNA

All of the clean tags were then searched against miRBase database(Release 22) to identify known (Species studied) miRNAs (exist miRNAs).

4.4.2 Identification of known miRNA

So far the miRNA sequences of some species were still not included in miRBase database. For those species the miRNAs alignment with other species was a dependable way to identify the known miRNAs.

4.4.3 Identification of novel miRNA

All of the unannotated tags were aligned with reference genome. According to their genome positions and hairpin structures predicted by software mirdeep2, the novel miRNA candidates were identified.

The default parameters of software mirdeep2 were as follows:

-c： input file is FASTA format

-h： parse to FASTA format

-i： convert RNA to DNA alphabet (to map against genome)

-j： remove all entries that have a sequence that contains letters other than a, c, g, t, u, n, A, C, G, T, U, or N.

-k <seq>： clip 3' adapter sequence

-l <int>： discard reads shorter than <int> nts

-m： collapse reads

-p <genome>：map to genome (must be indexed by bowtie-build). The genome string must be the prefix of the bowtie index. For instance, if the first indexed file is called h_sapiens_37_asm.1.ebwt then the prefix is h_sapiens_37_asm.

-q：map with one mismatch in the seed (mapping takes longer)

-s file：print processed reads to this file

-t file：print read mappings to this file

4.5 Small RNA annotation summary

After tags were annotated as mentioned previously, the annotation results were determined in this priority order: rRNA etc > exist miRNA > exist miRNA edit > known miRNA > repeat > exon > novel miRNA > intron.The tags that cannot be annotated as any of the above molecules were recorded as unann.

5 miRNA expression profiles

Total miRNA consists of exist miRNA, known miRNA and novel miRNA, based on their expression in each sample, the miRNA expression level was calculated and normalized to transcripts per million (TPM).

The formula is as follows:

$$TPM=Actual\ miRNA\ counts/Total\ counts\ of\ clean\ tags*10^6$$

In addition, the expression of exist miRNA, known miRNA and novel miRNA was also analysed individually.

5.1 miRNA Principal Component Analysis

Correlation analysis of two parallel experiments provides the evaluation of the reliability of experimental results as well as operational stability. The correlation coefficient between two replicas was calculated to evaluate repeatability between samples. The closer the correlation coefficient gets to 1, the better the repeatability between two parallel experiments.

5.2 miRNA Expression Pattern Clustering Analysis

The heatmaps of exist miRNA, known miRNA and novel miRNA were drew to display miRNA expression levels in different samples and to cluster miRNAs with similar expression pattern.

6 Differentially expressed miRNA (DE miRNA) Analysis

miRNAs differential expression analysis was performed by edgeR software between two different groups or samples.We identified miRNAs with a fold change ≥2 and P value <0.05 in a comparison as significant DE miRNAs.

7 Target gene Prediction

Based on the sequences of the exist miRNAs, known miRNAs and novel miRNAs, the candidate target genes were predicted as follows:

1) For animal samples, two softwares Miranda(Version 3.3a) and TargetScan(Version 7.0) were used to predict targets. The intersection of the results were more credible to be chosen as predicted miRNA target genes.

The default parameters of software Miranda were as follows:

1. The set score threshold is 140
2. The set energy threshold is -10 kcal/mol
3. Demand strict 5’ seed pairing
4. The gap-open penalty is -4.0
5. The gap-extend penalty is -9.0

The default parameters of software TargetScan were as follows:

The 2-8nt sequences which start from 5’ small RNA were choose as seed sequences to predict with 3’-UTR of transcripts.

2) For plant samples, the software patmatch(Version 1.2) was used to predict target genes.

The default parameters were as follows:

1. No more than four mismatches between sRNA \& target (G-U bases count as 0\.5 mismatches)
2. No more than two adjacent mismatches in the miRNA/target duplex
3. No adjacent mismatches in in positions 2\-12 of the miRNA\/target duplex (5\' of miRNA)
4. No mismatches in positions 10-11 of miRNA/target duplex
5. No more than 2\.5 mismatches in positions 1\-12 of the of the miRNA/target duplex (5\' of miRNA)
6. Minimum free energy (MFE) of the miRNA\/target duplex should be \>\= 74% of the MFE of the miRNA bound to it\'s perfect complement

8 Target gene functional enrichment Analysis

8.1 GO Enrichment Analysis

Gene Ontology (GO) is an international standardized gene functional classification system which offers a dynamic-updated controlled vocabulary and a strictly defined concept to comprehensively describe properties of genes and their products in any organism. GO has three ontologies: molecular function, cellular component and biological process. The basic unit of GO is GO-term. Each GO-term belongs to a type of ontology.

GO enrichment analysis provides all GO terms that significantly enriched in DEGs comparing to the genome background, and filter the DEGs that correspond to biological functions. Firstly all DEGs were mapped to GO terms in the Gene Ontology database (http://www.geneontology.org/), gene numbers were calculated for every term, significantly enriched GO terms in DEGs comparing to the genome background were defined by hypergeometric test. The calculating formula of P-value is:

$$P = 1 - \sum_{i=0}^{m-1} \frac{\binom{M}{i}\binom{N-M}{n-i}}{\binom{N}{n}}$$

Here N is the number of all genes with GO annotation; n is the number of DEGs in N; M is the number of all genes that are annotated to the certain GO terms; m is the number of DEGs in M. The calculated p-value were gone through FDR Correction, taking FDR ≤ 0.05 as a threshold. GO terms meeting this condition were defined as significantly enriched GO terms in DEGs. This analysis was able to recognize the main biological functions that DEGs exercise.

8.2 Pathway Enrichment Analysis

Genes usually interact with each other to play roles in certain biological functions. Pathway-based analysis helps to further understand genes biological functions. KEGG is the major public pathway-related database [12]. Pathway enrichment analysis identified significantly enriched metabolic pathways or signal transduction pathways in DEGs comparing with the whole genome background. The calculating formula is the same as that in GO analysis.

$$P = 1 - \sum_{i=0}^{m-1} \frac{\binom{M}{i}\binom{N-M}{n-i}}{\binom{N}{n}}$$

Here N is the number of all genes that with KEGG annotation, n is the number of DEGs in N, M is the number of all genes annotated to specific pathways, and m is number of DEGs in M. The calculated p-value was gone through FDR Correction, taking FDR $\leq$ 0.05 as a threshold. Pathways meeting this condition were defined as significantly enriched pathways in DEGs. The functional enrichment of both target genes of miRNAs in single samples and DE miRNAs in a compare group were carried out in our analysis.

9 Structural analysis of miRNA

9.1 miRNA cluster analysis

miRNA cluster is a group of tandem mirnas closely located in the genome. These miRNA are co-transcribed by one promoter, and they exist together in the form of polycistronic, which has a certain coordination effect and stronger biological effect. According to criteria for classification in miRbase, miRNAs located on a chromosome with an interval of less than 10 kb are defined as belonging to an miRNA cluster.

9.2 miRNA base editing analysis

There may be base editing in some positions of miRNA, which will change the seed sequence and the target gene. Through the sequence alignment of each sample miRNA with the existing miRNA mature body and its precursor, the miRNA with possible base mutation can be found out.

9.3 miRNA families analysis

The analysis of miRNA families was used to identify if the miRNAs were exist in other species. The analysis result was marked as “+” or “-” which refered to exist or non-exist, respectively.

9. Reference

[1] Robinson M D, McCarthy D J, Smyth G K. edgeR: a Bioconductor package for differential expression analysis of digital gene expression data[J]. Bioinformatics, 2010, 26(1): 139-140.
[2] Cui X , Xu S M , Mu D S , et al. Genomic analysis of rice microRNA promoters and clusters[J]. Gene, 2008, 431(1-2):61-66.
[3] Chen T , Xi Q Y , Ye R S , et al. Exploration of microRNAs in porcine milk exosomes[J]. BMC Genomics, 2014, 15(1):100.
[4] Cock P., et al. The Sanger FASTQ file format for sequences with quality scores, and the Solexa/Illumina FASTQ variants. Nucleic Acids Research, 38(6):1767-1771
[5] Langmead B, Trapnell C, Pop M, et al. Ultrafast and memory-efficient alignment of short DNA sequences to the human genome[J]. Genome Biol, 2009, 10(3): R25.
[6] Hafner, M., P. Landgraf, et al. (2008). "Identification of microRNAs and other small regulatory RNAs using cDNA library sequencing." Methods 44(1): 3-12.
[7] Ruby, J. G., C. Jan, et al. (2006). "Large-scale sequencing reveals 21U-RNAs and additional microRNAs and endogenous siRNAs in C. elegans." Cell 127(6): 1193-207.
[8] Calabrese, J. M., A. C. Seila, et al. (2007). "RNA sequence analysis defines Dicer's role in mouse embryonic stem cells". Proc Natl Acad Sci USA 104(46):18097-102.
[9] Zhang, Y., X. Zhou, et al. (2009). "Insect-Specific microRNA Involved in the Development of the Silkworm Bombyx mori." PLoS One 4(3): e4677.
[10] Allen, E., Z. Xie, et al. (2005). "microRNA-directed phasing during trans-acting siRNA biogenesis in plants." Cell 121(2): 207-21.
[11] Schwab, R., J. F. Palatnik, et al. (2005). "Specific effects of microRNAs on the plant transcriptome." Dev Cell 8(4): 517-27.
[12] Kanehisa, M., M. Araki, et al. (2008). "KEGG for linking genomes to life and the environment." Nucleic Acids Res. 36 (Database issue): D480-4. ";
