# Supplementary figures and images for: Sweeping analysis of transcript profile in dengue virus serotype 3 infection and antibody-dependent enhancement of infection
Source: Virulence. 2021 Nov 8;12(1):2764–76. doi: 10.1080/21505594.2021.1996072 (PMC8583062; doi:10.1080/21505594.2021.1996072)

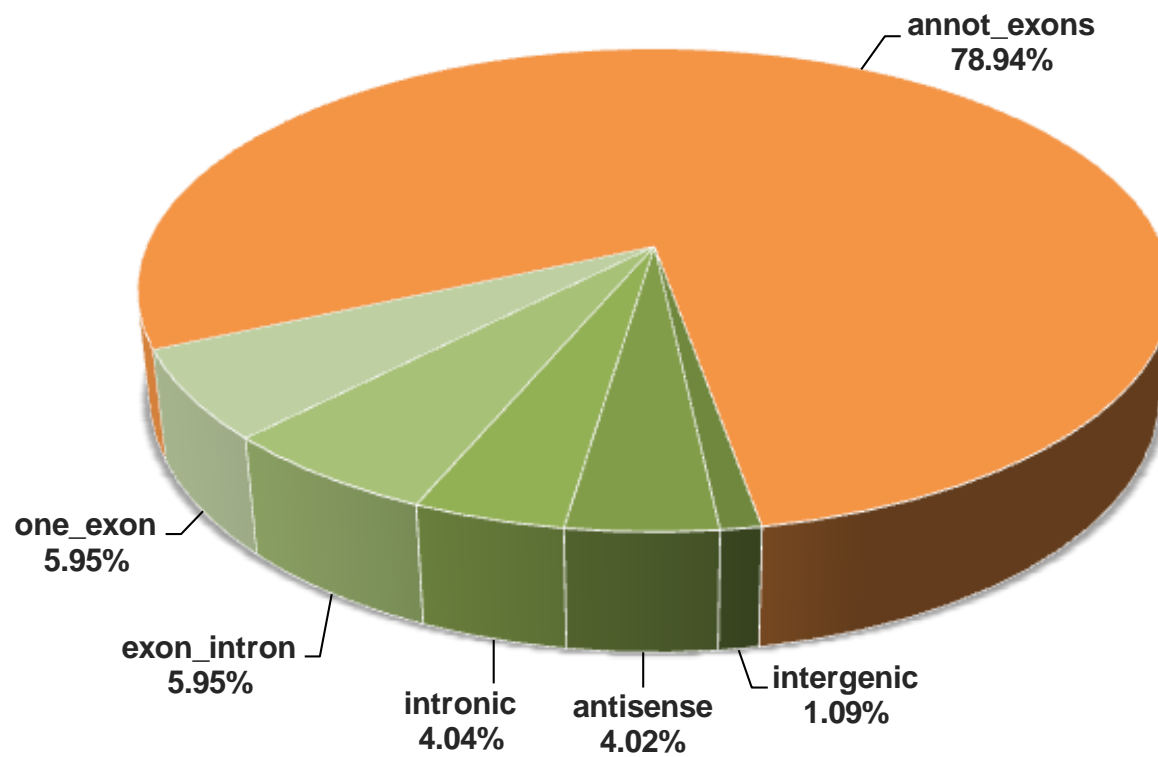

Supplement: Supplemental Material [file KVIR_A_1996072_SM1917.zip › Figure S1A.pdf]

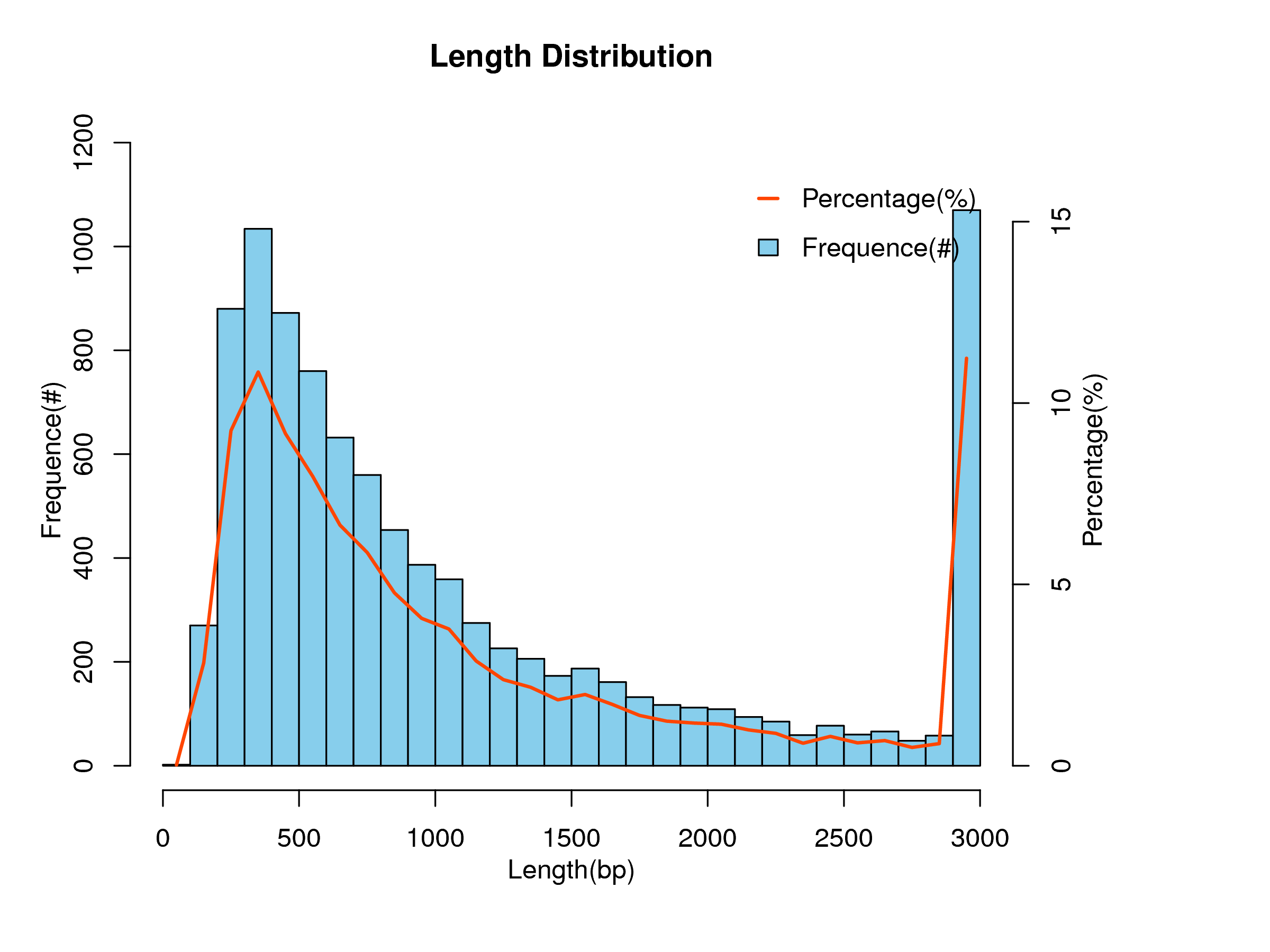

Supplement: Supplemental Material [file KVIR_A_1996072_SM1917.zip › Figure S1B.png]

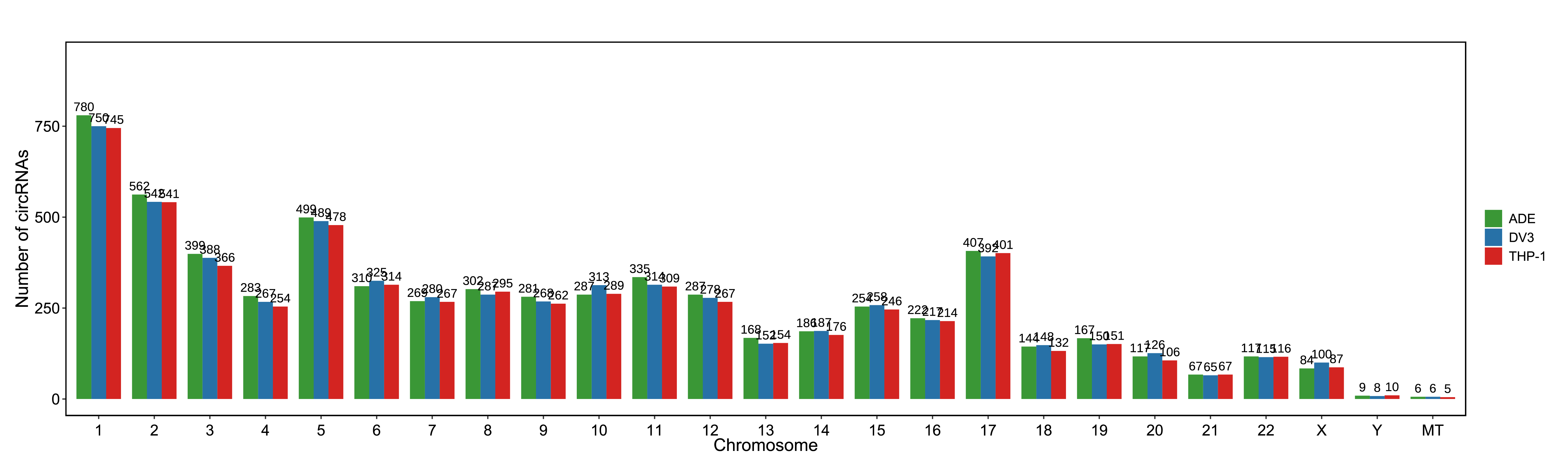

Supplement: Supplemental Material [file KVIR_A_1996072_SM1917.zip › Figure S1C.png]

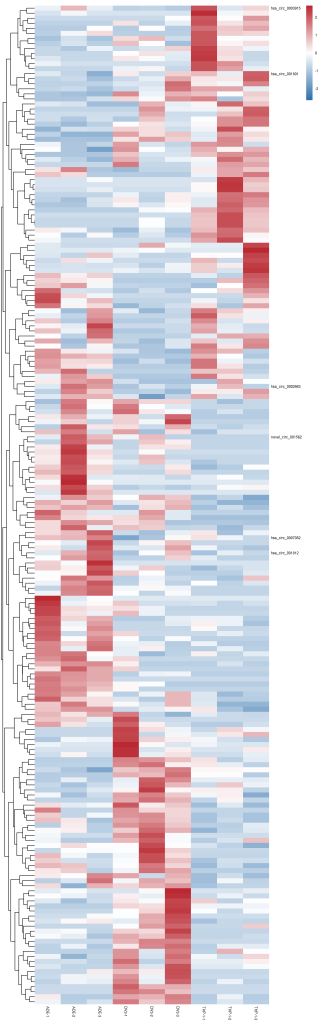

Supplement: Supplemental Material [file KVIR_A_1996072_SM1917.zip › Figure S1D.pdf]

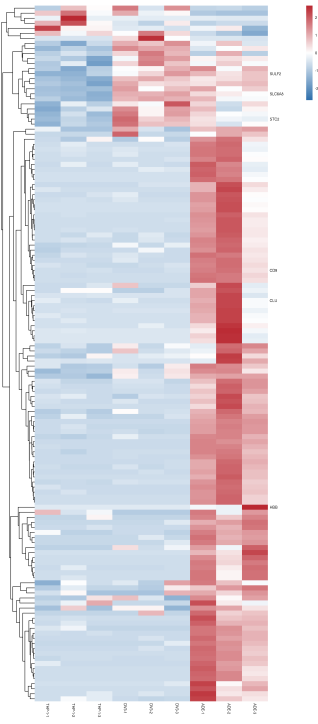

Supplement: Supplemental Material [file KVIR_A_1996072_SM1917.zip › Figure S2A.pdf]

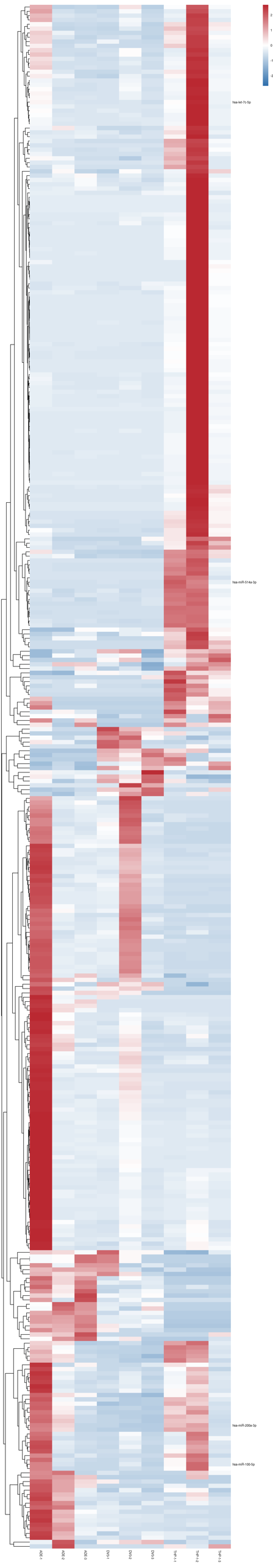

Supplement: Supplemental Material [file KVIR_A_1996072_SM1917.zip › Figure S2B.png]

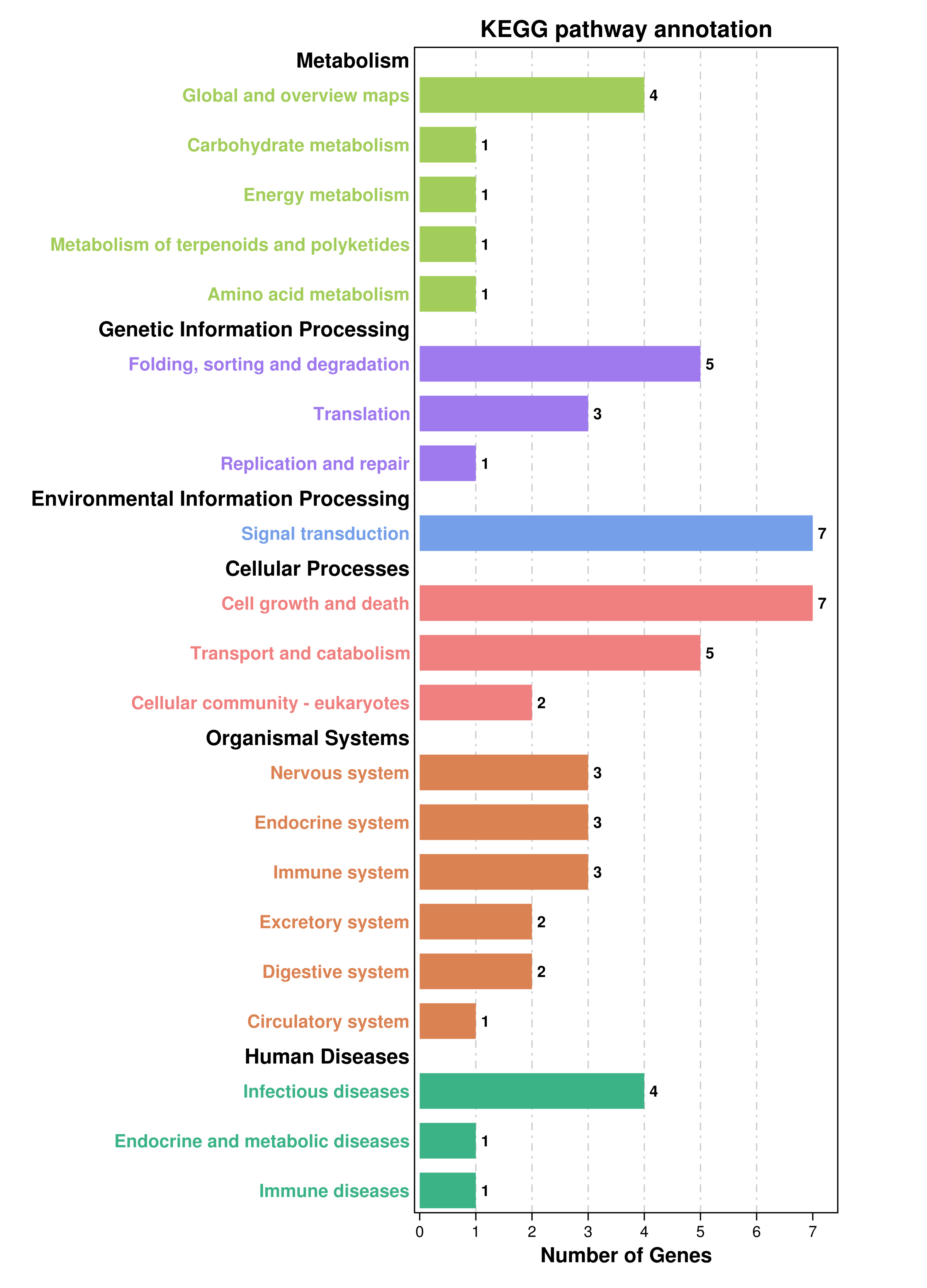

Supplement: Supplemental Material [file KVIR_A_1996072_SM1917.zip › Figure S3A.png]

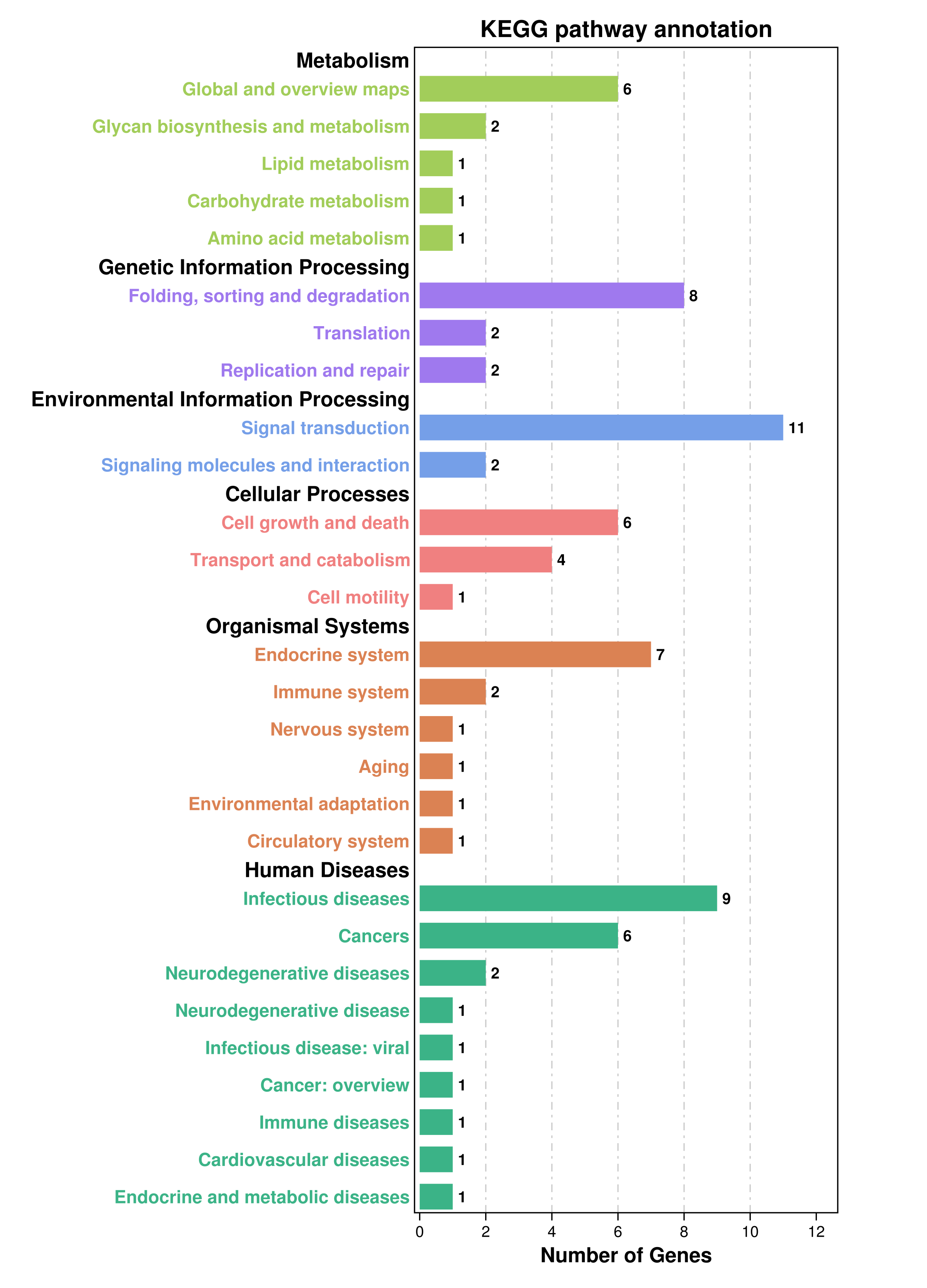

Supplement: Supplemental Material [file KVIR_A_1996072_SM1917.zip › Figure S3B.png]

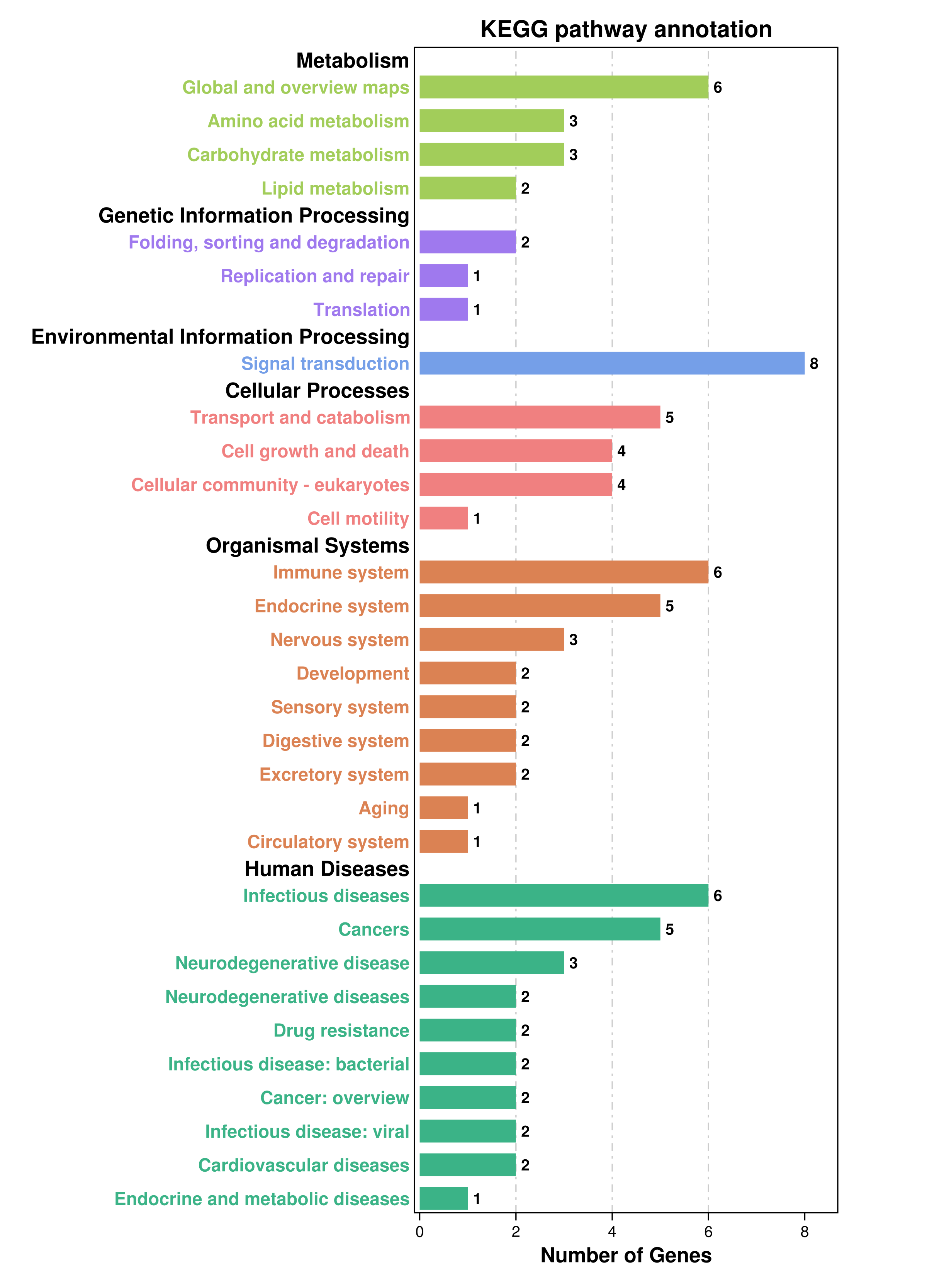

Supplement: Supplemental Material [file KVIR_A_1996072_SM1917.zip › Figure S3C.png]

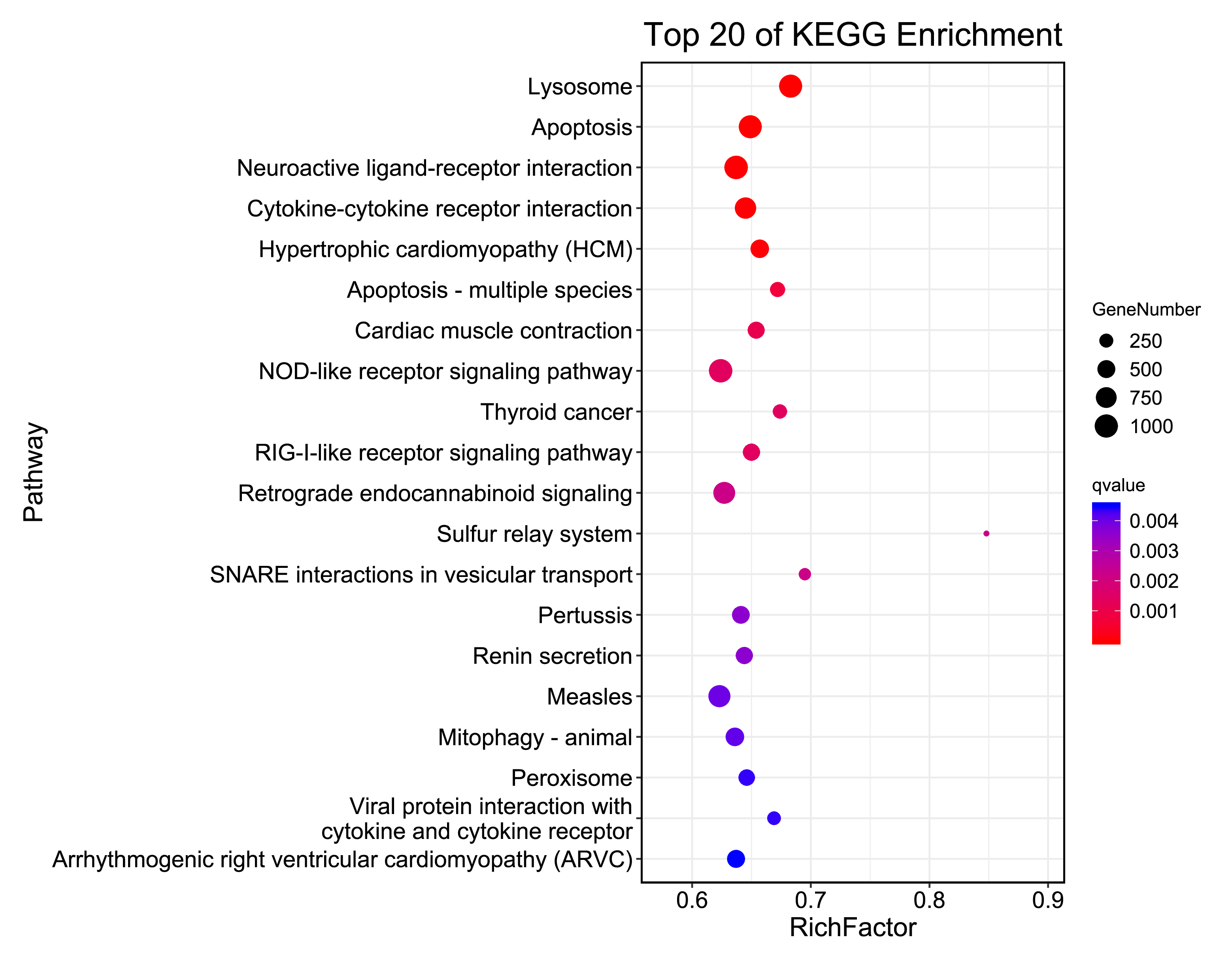

Supplement: Supplemental Material [file KVIR_A_1996072_SM1917.zip › Figure S3D.png]

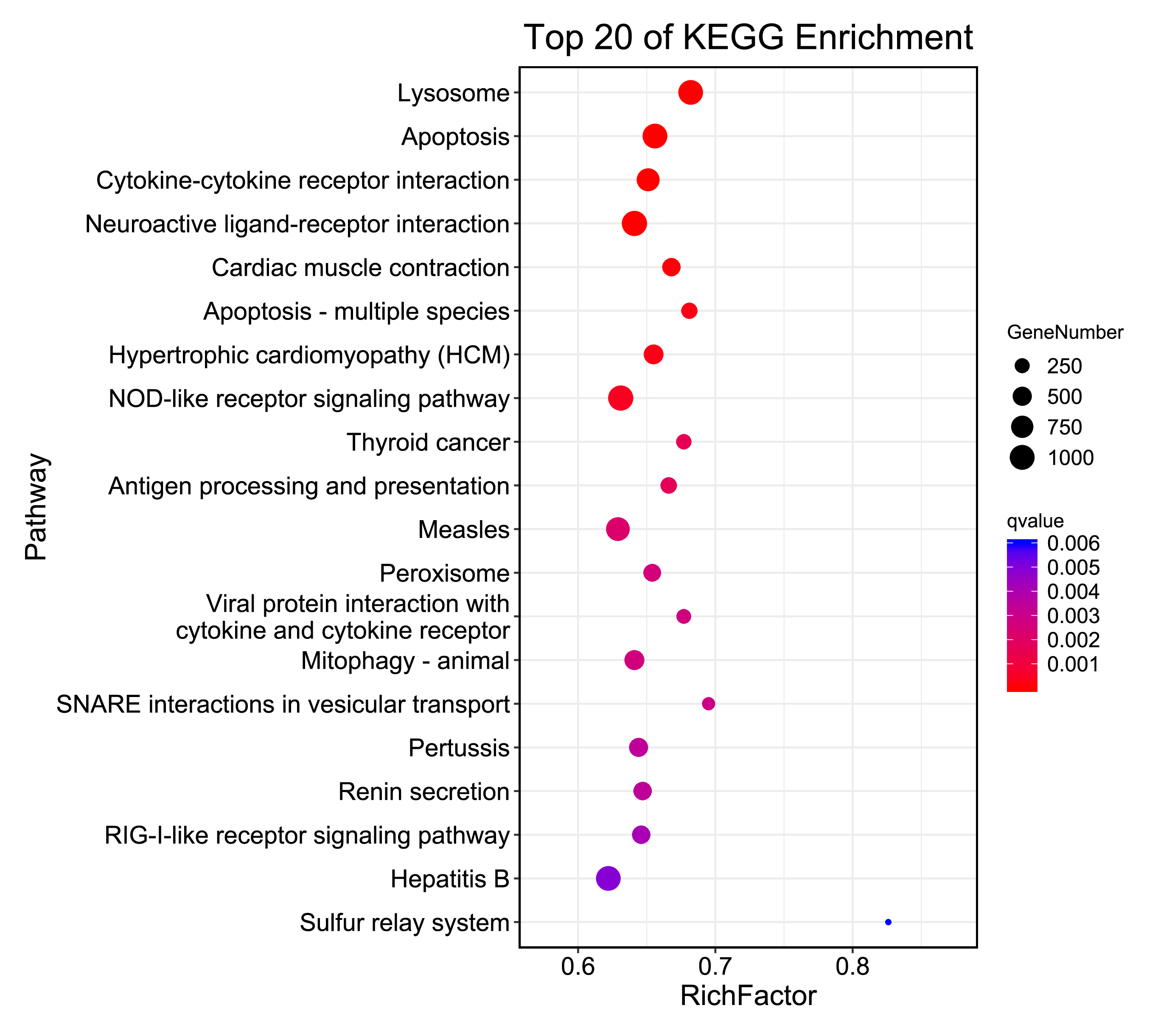

Supplement: Supplemental Material [file KVIR_A_1996072_SM1917.zip › Figure S3E.png]

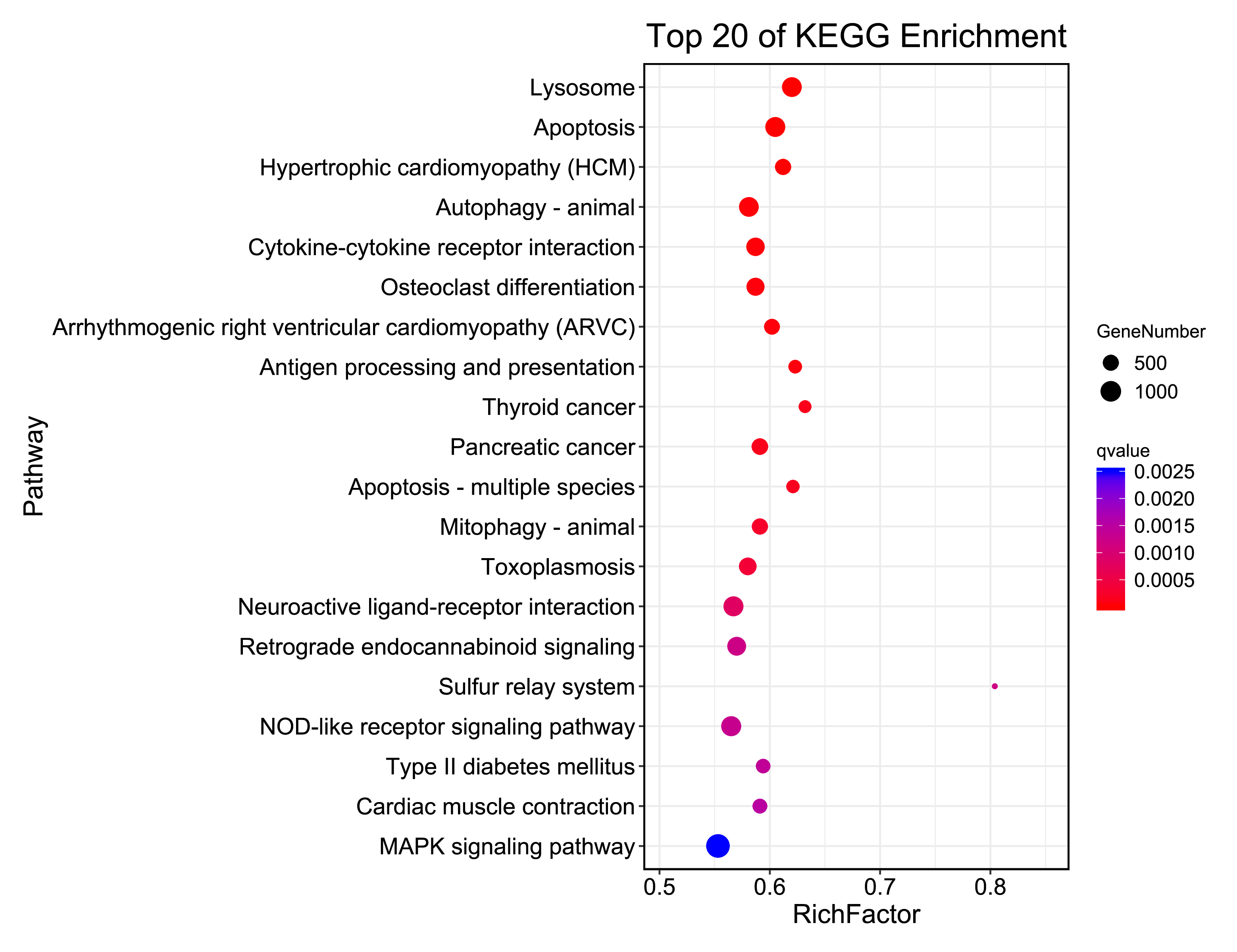

Supplement: Supplemental Material [file KVIR_A_1996072_SM1917.zip › Figure S3F.png]

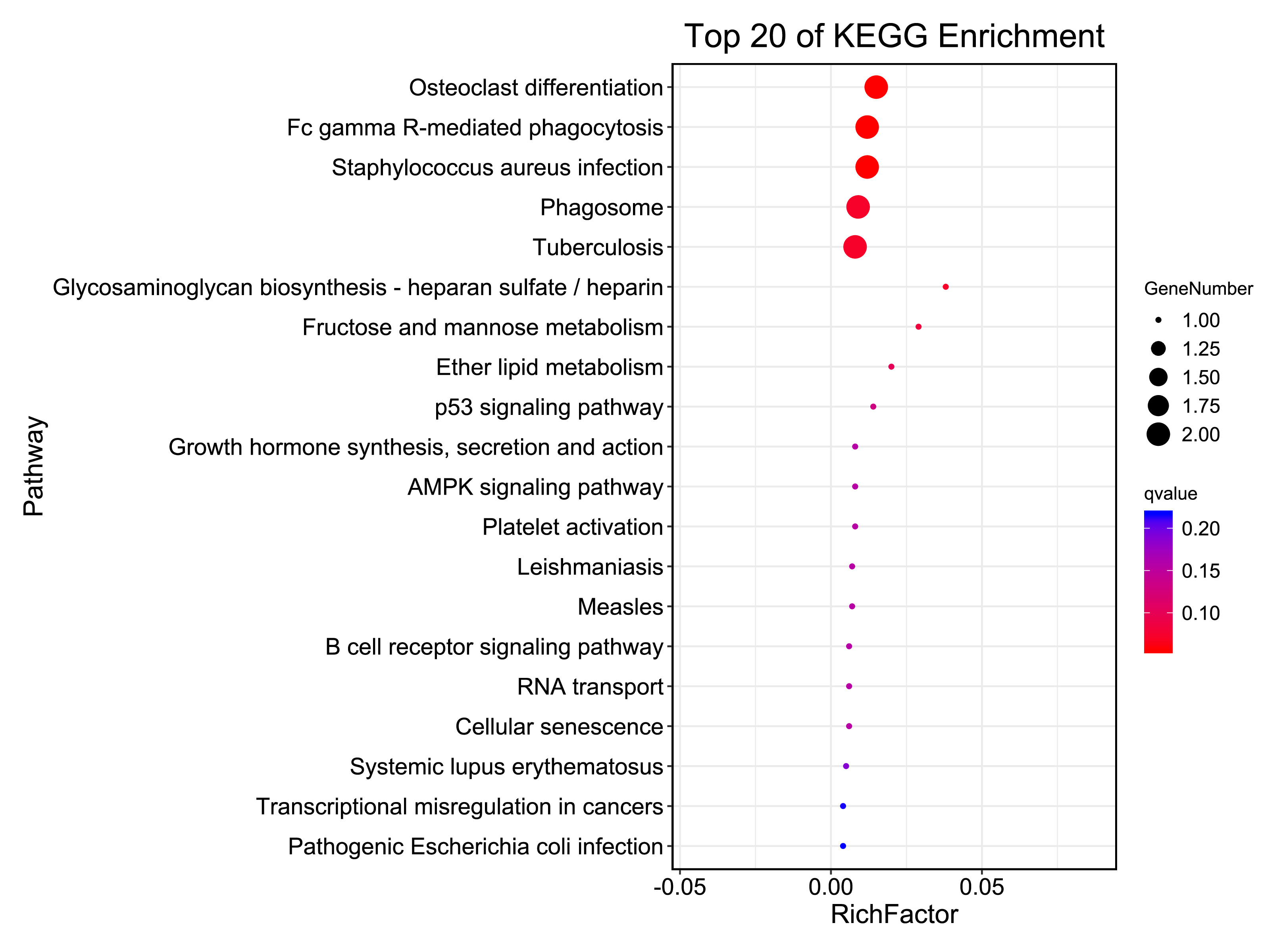

Supplement: Supplemental Material [file KVIR_A_1996072_SM1917.zip › Figure S3G.png]

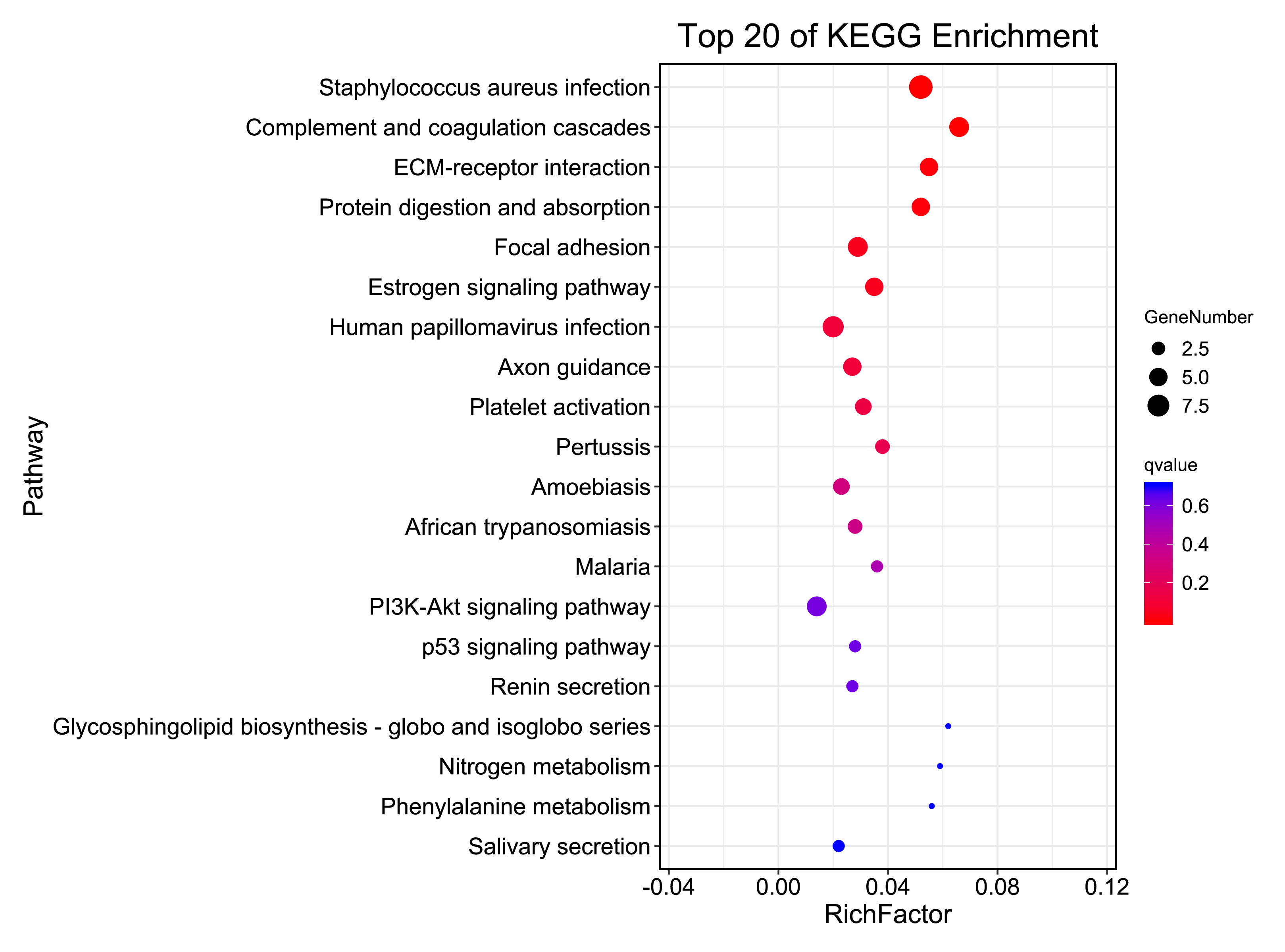

Supplement: Supplemental Material [file KVIR_A_1996072_SM1917.zip › Figure S3H.png]

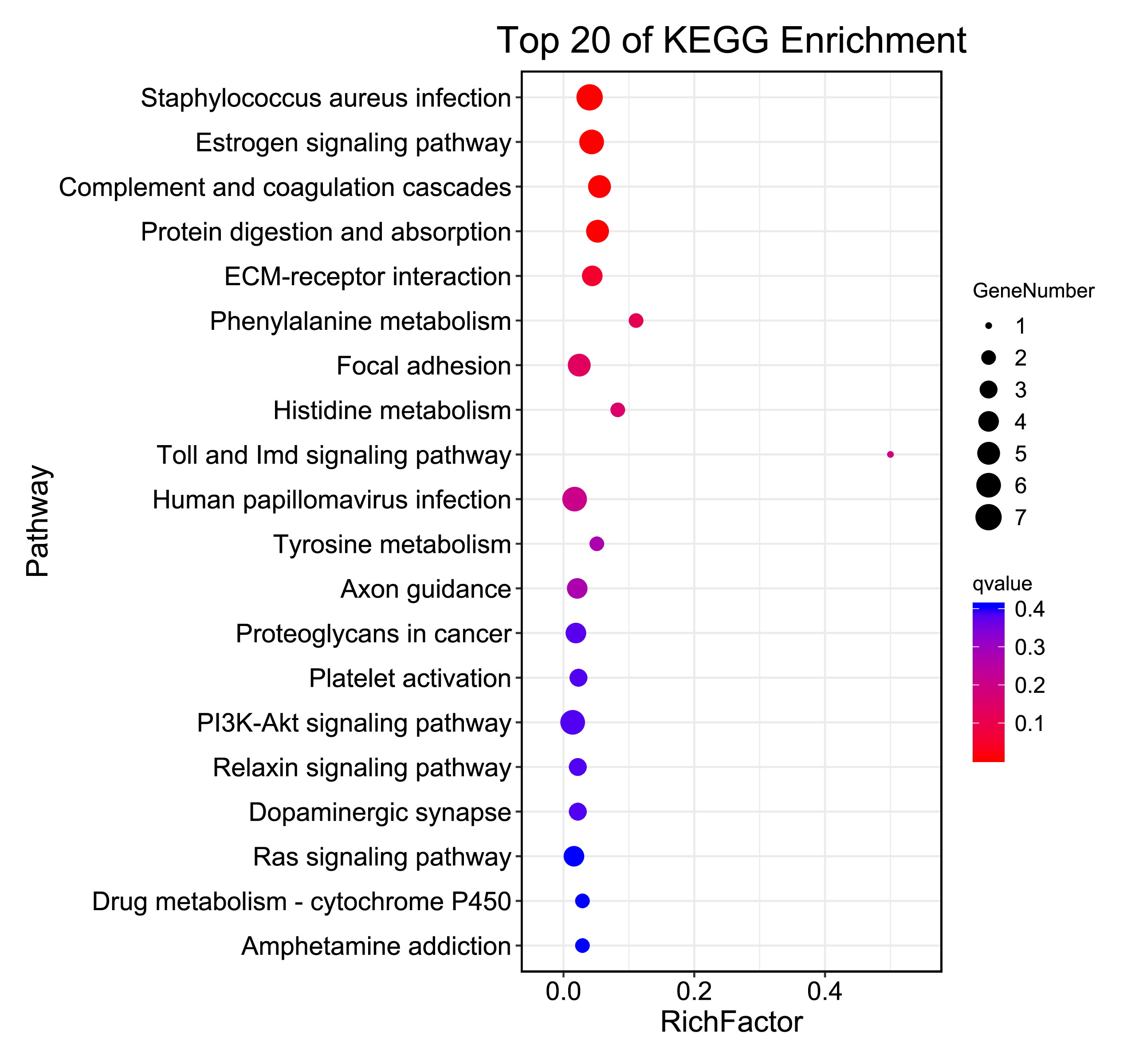

Supplement: Supplemental Material [file KVIR_A_1996072_SM1917.zip › Figure S3I.png]
