# Supplementary material for: Sweeping analysis of transcript profile in dengue virus serotype 3 infection and antibody-dependent enhancement of infection
Source: Virulence. 2021 Nov 8;12(1):2764–76. doi: 10.1080/21505594.2021.1996072 (PMC8583062; doi:10.1080/21505594.2021.1996072)
Supplement: Supplemental Material [file KVIR_A_1996072_SM1917.zip › Supplementary material 1.doc]

***Virulence***

***—— Supplementary material 1.***

**Sweeping analysis of transcript profile in Dengue virus serotype 3 infection and Antibody-Dependent Enhancement of Infection**

Mingwang Long^a,b,c^, Yue Pan^a,b,c^, Junying Chen^a,b,c^, Fan Jia^a,b,d^, Han Wang^a,b,c^, Daiying Li^a,b,c^, Kai feng^a,b,c^, Lingmei Yan^a,b,c^, Xiaodan Wang^a,b,c^, Xuelei Ning^a,b,e^, Lijuan Qiu^b^, Juan Zhang^a,b,d^, Qiangming Sun ^a,b,c^*

^a^Institute of Medical Biology, Chinese academy of Medical Sciences, and Peking Union Medical College, Kunming, Peoples Republic of China

^b^Yunnan Key Laboratory of Vaccine Research & Development on Severe Infectious Diseases, Kunming, PR China

^c^Yunnan Key Laboratory of Vector-borne Infectious Disease, Kunming, Peoples Republic of China

^d^Kunming Medical University, Kunming, Peoples Republic of China

^e^Yunnan University, Kunming, Peoples Republic of China

***Corresponding Author:**

Qiangming Sun, e-mail: [qsun@imbcams.com.cn](mailto:qsun@imbcams.com.cn)

**Figure legends**

**FIGURE S1. Characteristic analysis of de circRNAs.**

DE circRNAs annotation type **(A)**. DE CircRNAs length distribution **(B)**. Chromosome distribution of DE circRNAs **(C)**. Heatmap analysis **(D)** of DE circRNAs in THP-1 vs DENV-3, THP-1 vs ADE and DENV-3 vs ADE. The circRNA is indicated by a row and the sample is indicated by a column. Red indicates high expression and blue low expression.

**FIGURE S2. Differentially expressed mRNAs and miRNAs profiles.**

Heatmap analysis **(A)** of DE mRNAs in THP-1 vs DENV-3, THP-1 vs ADE, and DENV-3 vs ADE. The mRNA is indicated by a row and the sample is indicated by a column. Red indicates high expression and blue low expression. Heatmap analysis **(B)** of DE miRNAs in THP-1 vs DENV-3, THP-1 vs ADE, and DENV-3 vs ADE. The circRNA is indicated by a row and the sample is indicated by a column. Red indicates high expression and blue low expression.

**FIGURE S3. GO and KEGG analysis of DE RNAs.**

KEGG analysis of DE circRNAs in THP-1 vs DENV-3 **(A)**, THP-1 vs ADE **(B)**, DENV-3 vs ADE **(C)**. KEGG analysis of DE miRNAs in THP-1 vs DENV-3 **(D)**, THP-1 vs ADE **(E)**, and DENV-3 vs ADE **(F)**. KEGG analysis of DE mRNAs in THP-1 vs DENV-3 **(G)**, THP-1 vs ADE **(H)**, and DENV-3 vs ADE **(I)**.

**Table S1.** RT-qPCR primers used for the verification of DE circRNAs, DE miRNAs, and DE mRNA.

| **Gene ID** | Forward-primer(5’-3’) | Reverse-primer(5’-3’) |
| --- | --- | --- |
| novel_circ_001562 | CCACACTGAATGTCATAGTT | ACCCTTATTTCTTCCAAGTG |
| hsa_circ_001812 | ATTCCTAGATGGGCAAAGATG | GCCTTCTCATGATCAGCTC |
| hsa_circ_0003915 | AACCAAGTGCCAGGAGTT | GCGTGTTCTTCTCTGTTGT |
| hsa_circ_001801 | GAAGTCGAAGTCGTTCAA | CGTCTACGGTGTTTCTTT |
| hsa_circ_0002983 | AATCTACGCTGGTCCTCA | GGGCTTCGTTAAACTTCAC |
| hsa_circ_0007052 | CTTGTGGATGGACTTGCTA | GTAGGAGATAAAGGAGTGAGT |
| hsa-let-7c-5p | GCCGAGTGAGGTAGTAGGT | CTCAACTGGTGTCGTGGA |
| hsa-miR-100-5p | GCCGAGAACCCGTAGATCC | CTCAACTGGTGTCGTGGA |
| hsa-miR-514a-3p | GCCGAGATTGACACTTCT | CTCAACTGGTGTCGTGGA |
| hsa-miR-200a-3p | GCCGAGTAACACTGTCTGG | CTCAACTGGTGTCGTGGA |
| HBB | GGATGAAGTTGGTGGTGAG | TGAGGTTGTCCAGGTGAG |
| SLC6A8 | CCTCAGGTGTGGATAGATG | TGTAGCAGTTGTTGTTGAAG |
| STC2 | GCAGAATACAGCGGAGAT | AGGCGTCTTTGATGAATGA |
| SULF2 | GAGGACCAAGATGACAAGG | AGGATGTAGCACCGATGT |
| CLU | TCTTGTCTGTGGACTGTTC | AGGAGGTGTTGAGCATCT |
| CD9 | GTTCTTCGGCTTCCTCTT | GCAGTTCAACGCATAGTG |
| Plasmid-DENV-3-qRT-PCR | ACAGTTTCGACTCGG | CTTTCATTCTTCCCC |
| DENV-3-qRT-PCR | CCCCCGACTTCTTGAAGGTT | TGGATCACAGTTGGCGAAGA |
| GAPDH | TCGACAGTCAGCCGCATCT | CCGTTGACTCCGACCTTCA |
| **Gene ID** | Reverse transcription primer(5’-3’) | |
| hsa-let-7c-5p | CTCAACTGGTGTCGTGGAGTCGGCAATTCAGTTGAGaaccatac | |
| hsa-miR-100-5p | CTCAACTGGTGTCGTGGAGTCGGCAATTCAGTTGAGcacaagtt | |
| hsa-miR-514a-3p | CTCAACTGGTGTCGTGGAGTCGGCAATTCAGTTGAGtctactca | |
| hsa-miR-200a-3p | CTCAACTGGTGTCGTGGAGTCGGCAATTCAGTTGAGacatcgtt | |

A	B

C

D

A	B

**THP-1 vs DENV-3	THP-1 vs ADE	DENV-3 vs ADE**

**A	B	C**

**THP-1 vs DENV-3	THP-1 vs ADE	DENV-3 vs ADE**

**D	E	F**

**THP-1 vs DENV-3	THP-1 vs ADE	DENV-3 vs ADE**

**G	H	I**
