# Supplementary material for: Sweeping analysis of transcript profile in dengue virus serotype 3 infection and antibody-dependent enhancement of infection
Source: Virulence. 2021 Nov 8;12(1):2764–76. doi: 10.1080/21505594.2021.1996072 (PMC8583062; doi:10.1080/21505594.2021.1996072)
Supplement: Supplemental Material [file KVIR_A_1996072_SM1917.zip › Table S1..doc]

**Table 1.** RT-qPCR primers used for the verification of DE circRNAs, DE miRNAs and DE mRNA.

| **Gene ID** | Forward-primer(5’-3’) | Reverse-primer(5’-3’) |
| --- | --- | --- |
| novel_circ_001562 | CCACACTGAATGTCATAGTT | ACCCTTATTTCTTCCAAGTG |
| hsa_circ_001812 | ATTCCTAGATGGGCAAAGATG | GCCTTCTCATGATCAGCTC |
| hsa_circ_0003915 | AACCAAGTGCCAGGAGTT | GCGTGTTCTTCTCTGTTGT |
| hsa_circ_001801 | GAAGTCGAAGTCGTTCAA | CGTCTACGGTGTTTCTTT |
| hsa_circ_0002983 | AATCTACGCTGGTCCTCA | GGGCTTCGTTAAACTTCAC |
| hsa_circ_0007052 | CTTGTGGATGGACTTGCTA | GTAGGAGATAAAGGAGTGAGT |
| hsa-let-7c-5p | GCCGAGTGAGGTAGTAGGT | CTCAACTGGTGTCGTGGA |
| hsa-miR-100-5p | GCCGAGAACCCGTAGATCC | CTCAACTGGTGTCGTGGA |
| hsa-miR-514a-3p | GCCGAGATTGACACTTCT | CTCAACTGGTGTCGTGGA |
| hsa-miR-200a-3p | GCCGAGTAACACTGTCTGG | CTCAACTGGTGTCGTGGA |
| HBB | GGATGAAGTTGGTGGTGAG | TGAGGTTGTCCAGGTGAG |
| SLC6A8 | CCTCAGGTGTGGATAGATG | TGTAGCAGTTGTTGTTGAAG |
| STC2 | GCAGAATACAGCGGAGAT | AGGCGTCTTTGATGAATGA |
| SULF2 | GAGGACCAAGATGACAAGG | AGGATGTAGCACCGATGT |
| CLU | TCTTGTCTGTGGACTGTTC | AGGAGGTGTTGAGCATCT |
| CD9 | GTTCTTCGGCTTCCTCTT | GCAGTTCAACGCATAGTG |
| Plasmid-DENV-3-qRT-PCR | ACAGTTTCGACTCGG | CTTTCATTCTTCCCC |
| DENV-3-qRT-PCR | CCCCCGACTTCTTGAAGGTT | TGGATCACAGTTGGCGAAGA |
| GAPDH | TCGACAGTCAGCCGCATCT | CCGTTGACTCCGACCTTCA |
| **Gene ID** | Reverse transcription primer(5’-3’) | |
| hsa-let-7c-5p | CTCAACTGGTGTCGTGGAGTCGGCAATTCAGTTGAGaaccatac | |
| hsa-miR-100-5p | CTCAACTGGTGTCGTGGAGTCGGCAATTCAGTTGAGcacaagtt | |
| hsa-miR-514a-3p | CTCAACTGGTGTCGTGGAGTCGGCAATTCAGTTGAGtctactca | |
| hsa-miR-200a-3p | CTCAACTGGTGTCGTGGAGTCGGCAATTCAGTTGAGacatcgtt | |
